# Understanding and Controlling the Colloidal Stability of CdSe Nanoplatelets by Solvation Force Engineering

**DOI:** 10.1021/jacs.5c08392

**Published:** 2025-09-18

**Authors:** Shuai Chen, Nanning Petersen, Omar Valsson, Martin Girard, Hai I. Wang

**Affiliations:** † 28308 Max Planck Institute for Polymer Research, Mainz 55128, Germany; ‡ Department of Chemistry, 3404 University of North Texas, Denton, Texas 76201, United States of America; § Nanophotonics, Debye Institute for Nanomaterials Science, Utrecht University, Utrecht 3584 CC, The Netherlands

## Abstract

The interaction and steric stability of colloidal nanocrystals are determined by the interplay of various contributions, including van der Waals, dipole–dipole, and solvation forces. Recent simulations have unveiled that the solvation force dictates the colloidal stability of two-dimensional nanomaterials with no experimental validation. Here, we introduce optical-pump THz-probe spectroscopy (OPTP) as a novel approach to track the colloidal aggregation of two-dimensional nanoplatelets. We show that far below the saturation concentration previously determined by scattering methods, OPTP can already report nanoscale aggregations by sensitively probing the short-range free carrier photoconductivity arising from internanoplatelet electronic coupling. Combining the OPTP and molecular dynamics simulations allows us to (1) confirm that increasing the nanoplatelet base facet area results in enhanced solvation force and thus aggregation tendency, and (2) demonstrate that the attraction between the nanoplatelets increases with their chain length for *n*-alkane solvents. Finally, we extend our simulations to study the shape of the interaction that can be tuned via the isomer of the solvent molecules. Our results provide not only a new sensitive tool to probe the aggregation effects of semiconducting colloidal particles but also fundamental insights into the critical parameters to engineer colloidal stability.

## Graphical abstract

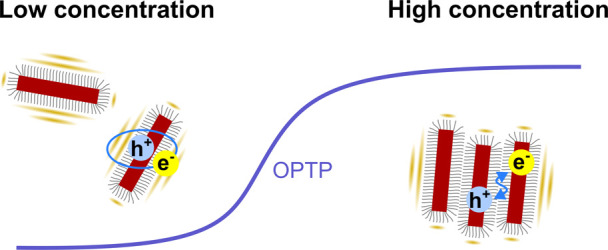

## 1 Introduction

Colloidal semiconductor nanocrystals (NCs) have attracted significant scientific attention due to their fascinating optoelectronic properties including size-dependent optical and electronic excitations. These properties are relevant for varied applications, e.g., in optoelectronics, biological imaging, and catalysis.^1–5^ A key motivation for employing NCs lies in their solution-processability, which is associated with low production costs. Understanding and controlling the colloidal stability is therefore critical for NCs processing and further device integration and optimization.

In general, the discrete nature of colloidal surfaces, and solvent molecules can strongly influence the interaction among colloidal NCs, and thus impact their colloidal stability.^6,7^ In apolar solvents, NCs are typically sterically stabilized via ligand shells.^8–10^ Their properties depend on many parameters including the ligand grafting density,^11^ the surface curvature,^12^ the ligand type,^13^ and the topology of the ligand shell.^14,15^ The nature of the solvent has also been demonstrated to affect the interaction between NCs.^16^

One generally assumes the ligand shell to be dilute and soft in the classical continuum models,^8,17–19^ where the transition between the ligand shell and the solvent is smooth. However, for dense shells, the classical continuum models fail. For example, Widmer-Cooper et al. have shown by simulations that the ligands of CdS nanorods in *n*-hexane form bundles at lower temperatures.^11^ These bundles can cause the formation of solvent layers away from the ligand-solvent interface, which in turn leads to solvation forces that impact the interaction of the CdS nanorods.^20^ In line with this result, we have recently demonstrated using simulations that solvation forces play a dominant role in these systems, by dictating aggregation and stacking of CdSe nanoplatelets (NPLs) in dispersions at room temperature.^21^

Our previous simulation findings provide a direct guide for modifying the interactions of NCs via solvation force “engineering” toward enhanced stability.^21^ While such forces govern pair interactions and drive NPLs stacking, probing the early formation of those nanoscale NPL clusters at very low concentrations remains challenging. For instance, small-angle X-ray scattering (SAXS) requires sufficiently large aggregates to detect, limiting its sensitivity to early stage clustering.^22,23^ Experimental access to the impact of solvation force on colloidal stability has also been limited. Furthermore, while we have predicted that the strength of the solvation forces can be tailored by tuning the ligand grafting density, the base facet area, and the ligand length, changing these parameters is not always practically easy. In order to achieve experimental tunability in both fundamental studies and device fabrication, more controllable parameters are required.

Here, we present a combined experimental and simulation effort via which we validate the role of solvation force in stabilizing CdSe nanoplatelet dispersions, and provide pathways to realize solvation force engineering. We first experimentally show that the CdSe nanoplatelets with larger base facet area turn to start aggregation at a lower concentration, in line with our previous simulation results, by carefully monitoring the onset of aggregation. Our study is enabled by using optical-pump THz-probe spectroscopy (OPTP), an all-optical technique which provides a sensitive probe to internanoplatelet electronic coupling and thus distance. By tracking concentration-dependent photoconductivity dynamics in CdSe nanoplatelet dispersions, we show that far below the critical aggregation concentration previously determined by scattering methods (e.g., by SAXS),^22^ OPTP can already report aggregations thanks to the enhanced free carrier conductivity in nanoscale aggregates. We then extend our experimental studies and molecular dynamics simulations to the comparison of different alkane solvents. Both experimental and simulation results consistently report that the attraction between the nanoplatelets increases with the chain length of *n*-alkane solvents. Finally, our simulations reveal the critical role of the shape of the solvent molecules in engineering the solvation force interaction.

## 2 Results and Discussion

### 2.1 Synthesis and Characterization of CdSe Nanoplatelets

We synthesize a series of zinc-blende 4 monolayer (ML) CdSe nanoplatelets with a fixed thickness of 1.4 nm corresponding to 4 Se layers and 5 Cd layers, capped with myristate ligands (see Sections 1.15 and 1.16 in the Supporting Information (SI) for synthesis details).^22,24,25^ By adjusting the growth time, four nearly square-shaped 4 ML CdSe nanoplatelets with tunable side lengths of ∼5, ∼10, ∼15, and ∼20 nm, corresponding to base facet areas ranging from 24, 119, 215, and 420 nm^2^ (see the transmission electron microscopy (TEM) studies as shown in Figure 1a–d).

**Figure 1:**
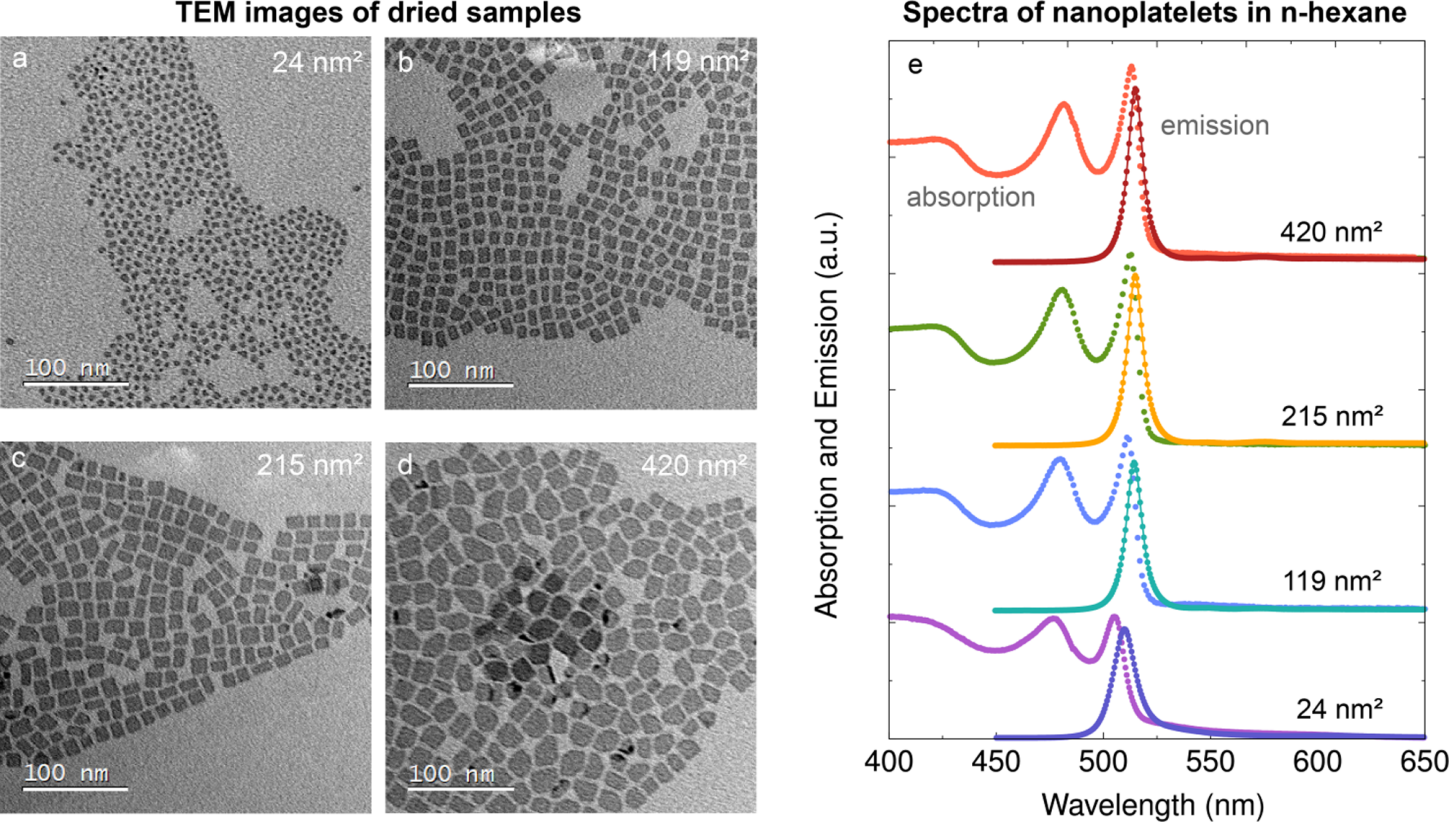
(a–d) show TEM images of the synthesized four monolayer thick CdSe nanoplatelets. The base facet area of the nanoplatelets in the samples is varied between ∼24 nm^2^ (a), ∼119 nm^2^ (b), ∼215 nm^2^ (c), and ∼420 nm^2^ (d). (e) Absorption and emission spectra for the four samples in *n*-hexane.

We measure the absorption and emission spectra of all samples, as presented in Figure 1e. We find sharp exciton peaks at around 480 and 512 nm, corresponding to the light and heavy hole transitions in CdSe, respectively.^26,27^ For the samples with around 119, 215, and 420 nm^2^ base facet area, we find no spectral shift in both absorption and photoluminescence (PL). This result is in line with previous reports indicating that the electronic structure of our nanoplatelets is predominantly controlled by the strong quantum confinement induced by the limited thickness^28^ rather than the lateral dimensions. A minor spectral shift of ∼7 nm is observed in the spectra of the 24 nm^2^ sample, as the side length of the base facets approaches the CdSe exciton Bohr radius of (4–6) nm.^29^

### 2.2 Facet Area Matters: Tracking Size-Dependent Nanoplatelets Aggregation Kinetics

While the lateral size plays a minor role in tuning optical and electronic properties of our nanoplatelets, in our previous work, we predicted that it strongly impacts colloidal stability: we showed that solvation forces are the dominant attractive interaction between CdSe nanoplatelets, while the core–core van der Waals interaction is negligible or much weaker (≤1 k_B_T for 225 nm^2^ facet area). Large facet areas of CdSe nanoplatelets lead to strong solvation forces between nanoplatelets and thus aggregation.^21^ To verify the increase in attraction with the base facet area, we employ OPTP spectroscopy to monitor how the concentration affects the nanoplatelet aggregation. The detection of nanoplatelet aggregation by OPTP lies in the fact that free carriers strongly absorb THz radiation and that changes in the aggregation state substantially affect free carrier generation probability as illustrated inFigure [Fig fig2]b. The THz field (*E*) accelerates free carriers to conduct, which in turn results in attenuation of the THz field. The photoinduced THz attenuation (Δ*E*) is proportional to the real part of photoconductivity Δσ_*R*_ ∝ Δ*E*.^26^ Furthermore, in addition to free charge carrier generation, photoexcitation of nanoplatelets produces also bound electron–hole pairs, i.e., excitons, which exhibit a distinctly different response in the THz frequency range. As charge-neutral species, excitons do not absorb the THz field; instead, they mainly induce a phase shift in the THz probe pulse due to their polarizability.^30^ The phase shift is proportional to the imaginary part of the photoconductivity Δσ_*I*_. Our recent study has demonstrated the power of THz spectroscopy in monitoring exciton formation and dynamics in aggregated CdSe nanoplatelet solids in thin films.^26^

**Figure 2:**
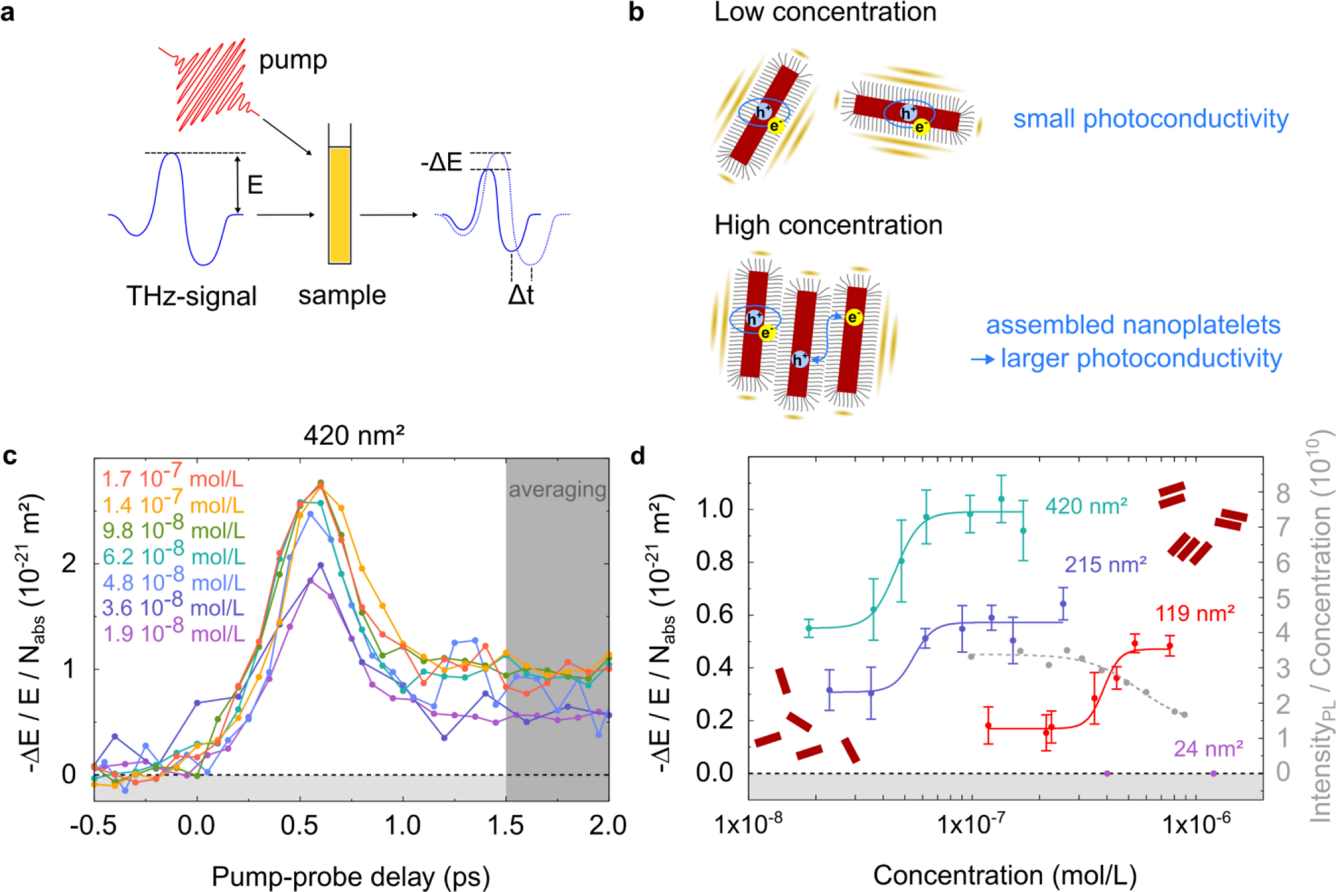
Investigation of charge carrier dynamics in CdSe nanoplatelets dispersion by THz spectroscopy. (a) shows a sketch of the optical-pump THz-probe spectroscopy setup. (b) shows a sketch of distinctively different charge carrier dynamics in single CdSe nanoplatelets at low concentrations (e.g., with exciton-dominated dynamics) and assembled nanoplatelets (with free-carrier contributed conductivity) at high concentrations. In (c), exemplary photoconductivity spectra for the nanoplatelets with the facet area of 420 nm^2^ in *n*-hexane are presented with real components. The photoconductivity is averaged between 1.5 and 2 ps after the pump pulse and summarized in (d). Concentration-dependent photoconductivity measurements were also performed for three additional nanoplatelet sizes for comparison. On the right *Y*-axis, the PL intensity normalized by concentration for the sample with an area of 119 nm^2^ is shown with gray dots.

To track the interactions among nanoplatelets in dispersion, we probe the concentration-dependent photoconductivity dynamics of CdSe nanoplatelet dispersions by OPTP for various facet areas. In Figure 2a, we show a sketch of the experimental setup (for details, see Section 1.1.1 and Figure S1 in the Supporting Information). In each experiment, we optically promote electrons in the nanoplatelets from the valence band to the conduction band by utilizing an ultrafast laser pulse (∼100 fs, 400 nm, 3.1 eV). The pump-induced change in the photoconductivity Δσ, is probed via a THz pulse with a duration of ∼1 ps. In Figures 2c and S6, we present concentration-dependent photoconductivity dynamics (Δσ ∝ $-\frac{\Delta E}{E}$ ) for CdSe nanoplatelets with a base facet area of around 420 nm^2^ in *n*-hexane. Within the concentration range we considered, we do not observe precipitation of the nanoplatelets by eye during our measurements. To make a fair comparison for all four nanoplatelet sizes with different concentrations, we normalize all the photoconductivity Δσ data to the absorbed photon density (*N*_abs_).

In our 4 ML CdSe nanoplatelets, pulsed excitation creates exciton-dominated charge species with large imaginary components. When the nanoplatelets form dimers or stacks, the electronic coupling between the nanoplatelets increases, resulting in increasing free carrier generation quantum yield in the system. As such, one would expect an increase in the photoconductivity Δσ_R_ with increasing concentration. This transition in the photoconductivity in the aggregation process can be measured by the OPTP experiments, which can report on the aggregation of nanoplatelets.

We summarize the size- and concentration-dependent real photoconductivity (average 1.5 to 2 ps) in the NPLs dispersion under the excitation photon energy of 3.1 eV for all the samples. As shown in Figure 2d, perfectly in line with the expectation, we observed a sigmoidal-like transition in the photoconductivity by increasing the nanoplatelet concentration. As we can see, for sufficiently large nanoplatelets (with facet areas of 119, 215, and 420 nm^2^), the sigmoidal-type transition in the concentration-photoconductivity plot can be persistently observed. Here we assign the transitions to the saturation concentrations, where the nanoplatelets begin to assemble. For the smallest-sized nanoplatelet, the photoconductivity dynamics are purely imaginary (Figure S7), indicating no aggregation occurring across the entire concentration range.

We note that the inferred aggregation concentration is much lower than that of scattering-type studies reported in the same colloidal system (e.g., ∼10^–8^ mol/L for THz study vs ∼10^–6^ mol/L for small-angle X-ray scattering).^22^ We attribute this discrepancy to the fundamentally different detection principles of the two techniques. OPTP does not directly measure interparticle distances, but it is highly sensitive to nanoscale free carrier generation and transport within nanoplatelet clusters.^26,31–33^ In our system, this sensitivity arises because clustering promotes internanoplatelet charge separation, producing free charge carriers capable of electrical conduction. In contrast, bare, well-dispersed single nanoplatelets exhibit optical and electrical responses dominated by “insulating” excitons, which do not contribute to current. Thus, OPTP serves as a novel and sensitive probe of nanoplatelet aggregation state through its response to electronic interactions. On the other hand, the size resolution of scattering characterizations relies on the interactions between the electromagnetic wavelength used and the size of aggregates. This is usually limited to a few to several hundred nm, depending on the wavelength of the probing beam.^34^

Our assignment of the nanoplatelet aggregation-induced sigmoidal photoconductivity transition is supported by two observations: (1) we investigate complementarily concentration-dependent PL emission (Figure 2d, right *Y* axis). In the diluted limit, the PL efficiency normalized to the concentration (η_PL_/*c*) is expected to show no dependence on the concentration (or, in other words, the PL efficiency increases linearly with concentration). With further concentration increase, η_PL_/c goes down. This can be rationalized as follows: the aggregation favors internanoplatelet charge transport (CT) or energy transfer (ET, e.g., via the Förster-mechanism). The enhanced CT or ET reduces PL efficiency, as they result in charge separation, increasing their nonradiative recombination via defects (as charges can map out much larger space to encounter defects).^35,36^ Our experimental result aligns nicely with the expectation and previous reports. More importantly, the critical concentration at which η_PL_/*c* starts to decrease is close to the saturation concentration quantified by OPTP measurements (Figure 2d, right *Y* axis). (2) the critical concentration for aggregation is reduced with the nanoplatelets’ base facet area: i.e., the larger the nanoplatelets, the easier they aggregate. This is in agreement with our prediction that the attraction between the nanoplatelets increases with the base facet area,^21^ and thus the nanoplatelets begin to aggregate at smaller nanoplatelet concentrations.

We note that the base level of photoconductivity at low concentrations increases with the base facet area. We do not yet fully understand the physical origin of this effect. This might be related to (1) some degree of NPL aggregates during sample preparation which did not get fully filtered out. These residual aggregates may contribute to photoconductivity via charge separation. Larger facet areas promote stronger aggregation, which in turn can lead to higher photoconductivity; (2) intrinsic intra-nanoplatelet free carrier generation by energetic pulse excitation. To investigate the effect of pump photon energy on photoconductivity at low concentration, we compared the conductivity dynamics following 2.3 eV resonant excitation with those induced by 3.1 eV pumping. As shown in Figure S7c, the photoconductivity of low-concentration samples decreases by approximately 30% for the largest NPLs (∼20 nm side length) when excited at 2.3 eV compared to 3.1 eV. This result suggests that intrinsic free-carrier generation via hot exciton dissociation can occur in samples with sufficiently large facet areas. Overall, our findings indicate that at least 70% of the low-concentration photoconductivity originates from aggregation effects, while at most 30% arises from hot exciton dissociation within individual flakes.

### 2.3 Solvent Nature Matters: Tracking Solvent Length-Dependent Nanoplatelets Aggregation

In the next step, we study the effect of different solvents on the nanoplatelet aggregation. We begin by comparing *n*-hexane, *n*-octane, *n*-decane, and *n*-dodecane, by measuring the nanoplatelet concentration-dependent photoconductivity (see Figure 3a, and all corresponding dynamics in Figures S4, S9, S10, and S11). For all four solvents, we find again a sigmoidal transition. Interestingly, the photoconductivity in the high concentration limit remains unchanged for all four solvents. This leads us to conclude that the average distance between nanoplatelets within the aggregates is similar across all solvent conditions. The saturation concentration, on the other hand, decreases as the *n*-alkane length increases; this can be attributed to an increase in the attraction between the nanoplatelets.

**Figure 3:**
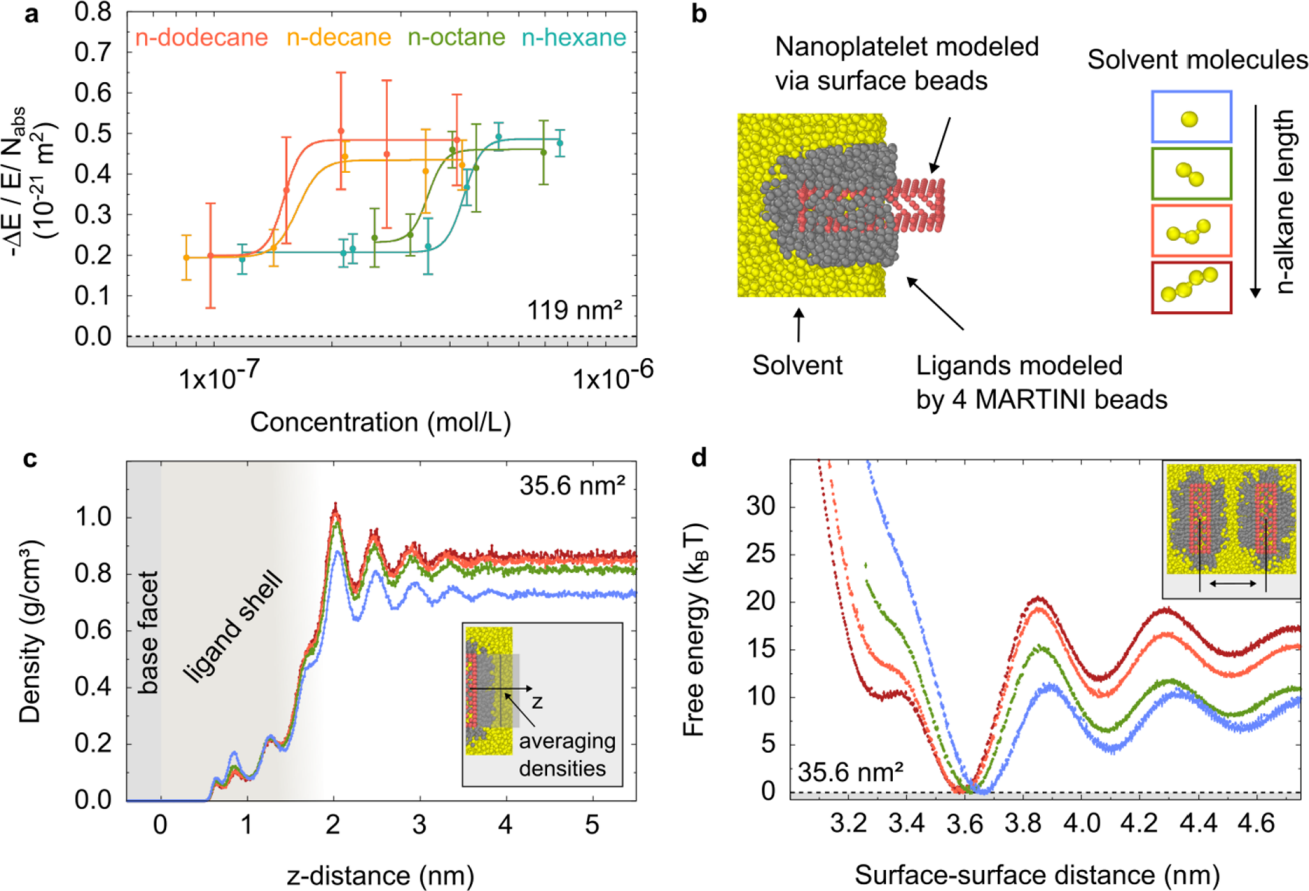
Effect of the *n*-alkane solvent length. (a) shows the experimentally measured photoconductivity. The base facet area of the nanoplatelets in this sample is around 119 nm^2^. The panels (b–d) show simulation results for nanoplatelets with 35.6 nm^2^ base facet area. (b) shows the nanoplatelet setup with the MARTINI model, as well as snapshots of the four solvent molecule types. Generally, one MARTINI bead represents an alkyl-chain with 4 CH_*x*_ groups. (c) displays the averaged solvent densities away from the base facet of single nanoplatelets (see depiction in the inset), while (d) shows calculated free energy curves that describe the base facet-to-base facet interaction of two nanoplatelets in the considered *n*-alkane solvent types (see inset).

To provide physical insight into our experimental results, we run molecular dynamics simulations to study how the solvation forces depend on the *n*-alkane length. Briefly, we model the CdSe nanoplatelets as rigid objects, to which ligands are grafted (see Figure 3b). Thereby, we neglect the core–core van der Waals attraction. However, we explicitly describe the solvent. Here, we apply the MARTINI force field to describe the ligand and solvent molecules, as well as their interactions. We consider solvent molecules with a length between one and four C_1_ MARTINI beads. This covers the range of *n*-alkanes that are liquid at room temperature (*n*-pentane to *n*-hexadecane). We note that the melting point of the *n*-alkane solvents increases with the chain length. The melting point of *n*-hexadecane is already close to room temperature (18 °C). Also, the viscosity increases with the chain length (1.3 mPa·s for *n*-dodecane vs 3 mPa·s for *n*-hexadecane.

Our setup is well equipped to describe trends, e.g., the effect of increasing solvent length. However, we should note that such a coarse grained description does not cover all features of a fully atomistic description, or a system with a lower level of coarse graining. Therefore, our description with the MARTINI force field is rather qualitatively than quantitatively. A more detailed description of our model can be found in the SI (Section 2.1), and in our previous publication.^21^

Solvation forces are correlated to the changes within the solvent structure as two surfaces approach each other.^37^ Accordingly, we investigate the solvent density away from the base facet surface of the simulated nanoplatelets (Figure 3c). Close to the ligand-solvent interface, the solvent density oscillates, which is a consequence of the solvent structuring. The amplitude of this density oscillation increases with solvent length. As two nanoplatelets approach each other, the solvent structure changes, which leads either to a decrease or an increase of the free energy.^21^

In Figure 3d, we show free energy profiles for the pair interaction of two nanoplatelet base facets in the different *n*-alkane solvents, see Figure S20 in the Supporting Information for the full curves for 1 and 2 solvent beads. At the free energy minima, we find an integer number of well-defined solvent layers, including no solvent layers at the first free energy minimum, which corresponds to the global minimum in our previous publication^21^ (compare with density profiles at the free energy minima, see Figures S18, S19, S20, and S21). For *n*-alkanes, the oscillation period remains independent of the molecule length.^37,38^ However, the amplitudes of the density oscillation, as well as the amplitudes of the free energy oscillation increase with the chain length.

As a first approximation, the solvation forces and their corresponding free energy curve can be described as follows:^37^

$$G\left(d\right)\approx G_{0}\cos\left(\frac{2\pi D}{d}\right)e^{-D/d}$$

where *d* is the effective diameter of the solvent molecules, and *D* is the surface–surface distance. The amplitude *G*_0_ describes the strength of the interaction. Hence, the free energy difference between the first minimum and the first maximum is a good measure for the strength of the solvation forces. This is also what we have found in our previous publication for the effect of different facet areas, ligand lengths, and ligand grafting densities.^21^ Consequently, we conclude here in agreement with previous studies on solvation forces^37,38^ that the attraction increases with the solvent *n*-alkane chain length.

We find that the ligand molecules rearrange themselves slightly as the nanoplatelets approach each other. However, the dependence on the solvent type is rather weak (see Figure S21). To ensure that this is not an effect of the coarse graining in our model, we also simulate the ligand and solvent densities using an unified atom model (TraPPE-UA, see Section S2.1). We find very similar density profiles, in which the ligand density does not change significantly (see Figure S19). We attribute the independence of the ligand shell densities of the solvent type to the exceptionally dense ligand shell of CdSe nanoplatelets (5.4 ligands/nm^2^).^21^

We note that electrostatic forces, such as the core–core van der Waals attraction, would decrease in longer chain *n*-alkane solvents, since the dielectric constant increases. Therefore, these results highlight the dominant role of the solvation forces in the CdSe nanoplatelet system.^21,39^ We can conclude that the increase in the solvation forces most likely leads to an increase in the attraction between the nanoplatelets and therefore to a decrease in the measured saturation concentrations.

In the next step, we explore chemical topology as a means of engineering the shape of the interaction between the nanoplatelets. In order to do so, we turn to isomers of octane as our model system (see Figure 4a). As the MARTINI coarse-grain models cannot distinguish the different isomers, we turn to a united-atom force field (TraPPE-UA).

**Figure 4:**
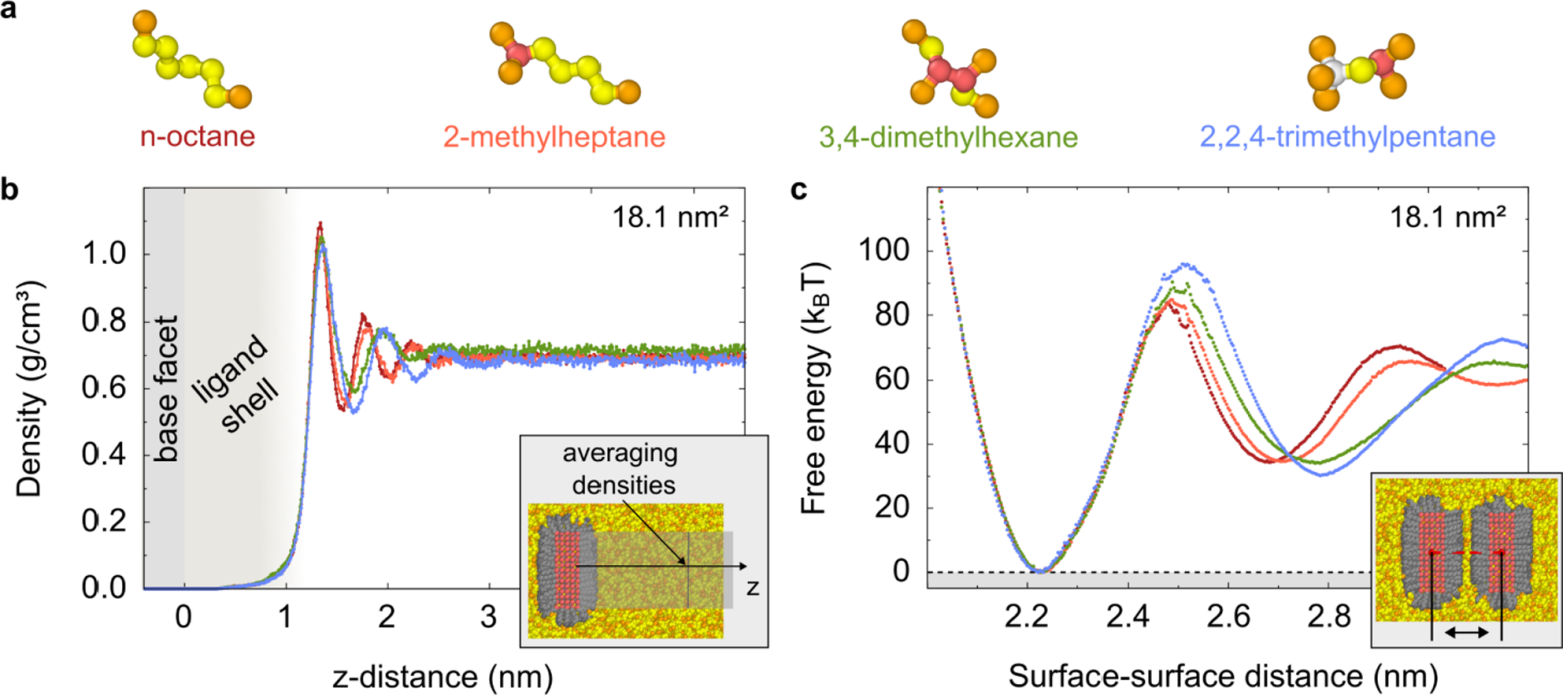
Comparison of octane isomers within the TraPPE-UA setup. Snapshots of the molecules are shown in (a). (b) displays the averaged solvent densities away from a base facet of single nanoplatelets with 18.1 nm^2^ base facet area, while (c) shows calculated free energy curves that describe the base facet-to-base facet interaction of two nanoplatelets in the considered solvent types. In this setup, ligands are modeled via 8 united atom beads.

Unlike linear chains, octane isomers cannot lie flat on the interface, and therefore appear to be bulkier than their linear counterparts. Hence, the oscillation period of the solvent density (see Figure 4b) increases as the “bulkiness” increases. These changes are also reflected in the free energy profiles (see Figure 4c), with increasing oscillation periods for isomers.

For all of isomers, the first minimum of the free energy lies at ∼3.44 nm. The free energy curves are nearly identical up to a separation of ∼3.65 nm. This goes hand in hand with nearly the identical ligand densities at the first minimum, where the solvent densities are nearly zero (Figure S24). Away from ∼3.65 nm, the free energy curves split. The peak of the first maximum shifts to larger distances, and the free energy value increases with the branching of the solvent molecules.

We relate both changes to the effective size of the isomers. Let us consider two hard flat surfaces and spherical solvent molecules. There must be a certain distance between the surfaces before the molecules get between them (see sketch in Figure S25 in the Supporting Information). Therefore, the distance increases with the size of the solvent molecules. Furthermore, the free energy increases due to the excluded volume respectively depletion effect Δ*G* ∝ – Δ*V*.^37,39^ This simplified model neglects important properties like the softness of the ligand shell, the different flexibility and interaction of the isomers, and that the solvent molecules will not move all at once into the volume between the ligand shells. However, if we assume that the effective size of the solvent molecules increases with the branching, the model illustrates the fundamental effect.

The second minima also shift to larger distances due to the increased effective size. This is also visible in the densities (Figure S24). However, there is no simple relationship between the densities and the free energy difference between the first and second minima. For example, the ligand and solvent density curves of 3,4-dimethylhexane and 2,2,4-trimethylheptane are nearly identical at the first and second minimum (Figure S24). Nevertheless, the free energy difference varies. This example illustrates that the free energy curves are not only determined by the densities, but also by the excluded volume effect, and the arrangement and interaction of the ligand and solvent molecules.

For the *n*-alkane solvents, we argued that the free energy difference between the first minima and first maxima is a good measure for the attraction between the nanoplatelets. Applying this rule of thumb here, we conclude that the attraction between the nanoplatelets increases from *n*-octane to 2,2,4-trimethylheptane with increased branching. However, we would like to note that the damping of the oscillation changes. Therefore, this is a rude approximation. Also, this is not a general trend. For example, Wang et al. have shown that solvation forces between flat, uncoated hard surfaces are stronger in *n*-decane than in 2,2-dimethyloctane because of structural changes in the configuration of the solvent molecules.^40^

In experimental solution processing, the solvent is often evaporated. The final structure arises from a competition of two rates: assembly and evaporation. The former is tightly related to free energy profiles, which we can control by the choice of the solvent type or isomer. The vapor pressure, controlling evaporation rate is however strongly affected by bulkiness; consider for instance octane (1.5 kPa) versus 2,2,4-trimethlypentane (5.5 kPa). Solvent length and bulkiness therefore provide orthogonal control over assembly and evaporate rates, respectively. For specific applications, an appropriate solvent can therefore be engineered by careful selection of length and bulkiness.

## 3 Conclusions

We report nanoscale aggregation effects in the low concentration end (in comparison to the scattering-type studies) by sensitively probing the short-range free carrier photoconductivity. Combined with molecular simulation, our ultrafast photoconductivity reports critical roles of the nanoplatelet base facet area and the solvent nature in controlling the solvation forces and colloidal stability of semiconducting CdSe nanoplatelets. Furthermore, our results show that solvation forces engineering offers interesting prospects for tuning nanoplatelet dispersion stability toward device fabrication. Moreover, OPTP spectroscopy can serve as a powerful technique to probe aggregation dynamics in a wide range of colloidal systems, providing a deeper understanding of colloidal stability and functional properties relevant to applications in optoelectronics, biological imaging, and catalysis.

## Supplementary Material



## Notes

Details for the OPTP, TEM, UV–vis, PL spectra, sample preparation, simulation technical, and additional simulation results (PDF)
