# Supplementary material for: Understanding and Controlling the Colloidal Stability of CdSe Nanoplatelets by Solvation Force Engineering
Source: J Am Chem Soc. 2025 Sep 18;147(39):35347–54. doi: 10.1021/jacs.5c08392 (PMC12498406; doi:10.1021/jacs.5c08392)
Supplement: Supplementary file 1 [file ja5c08392_si_001.pdf]

Supporting Information

Understanding and Controlling Colloidal Stability of CdSe Nanoplatelets by Solvation Force Engineering

Shuai Chen,[1,#] Nanning Petersen,[1,#] Omar Valsson, [2*] Martin Girard,[1,*] and Hai I. Wang[1,3*]

1. Max Planck Institute for Polymer Research, Mainz 55128, Germany

2. Department of Chemistry, University of North Texas, Denton, TX 76201, United States of America

3. Nanophotonics, Debye Institute for Nanomaterials Science, Utrecht University, Utrecht 3584 CC, The Netherlands

# Shuai Chen and Nanning Petersen contributed equally to this work

Corresponding E-mail: omar.valsson@unt.edu; martin.girard@mpip-mainz.mpg.de; h.wang5@uu.nl

Contents

1 Experiments 3

1.1 Methods 3

1.1.1 Optical-pump terahertz-probe spectroscopy (OPTP) 3

1.1.2 Transmission Electron Microscopy (TEM) 4

1.1.3 Ultraviolet-visible (UV-vis) and Photoluminescence (PL) spectroscopy 4

1.1.4 Chemicals 4

1.1.5 Synthesis of Cadmium myristate [Cd(myristate)$_2$]. 5

1.1.6 Synthesis of 4 Monolayer (4 ML) CdSe nanoplatelets 5

1.1.7 Concentration-dependent PL emission measurements 6

1.2 Additional experimental results 7

2 Simulations 10

2.1 Technical details 13

2.1.1 Model system setups 13

2.1.2 Infinite facet setup 15

2.1.3 Choice of the nanoplatelet dimensions and ligand length 16

2.1.4 Free energy calculation 17

2.2 Additional simulation results 21

2.2.1 Facet area 21

2.2.2 Ligand length 23

2.2.3 n-alkane solvent length 24

2.2.4 Isomers of octane 28

1 Experiments

1.1 Methods

1.1.1 Optical-pump terahertz-probe spectroscopy (OPTP)

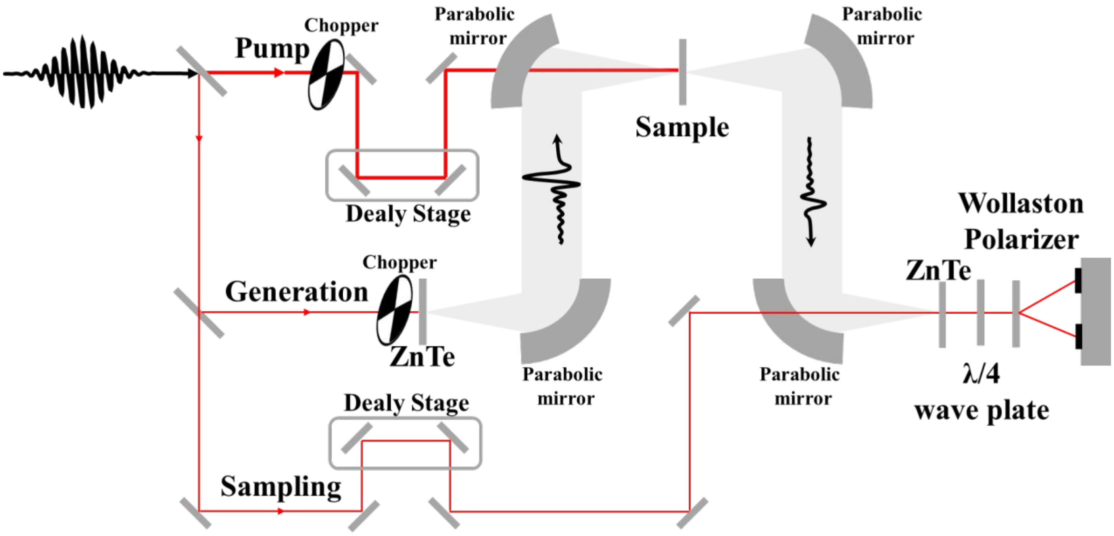

Figure S1 Schematic diagram of the OPTP setup used in this project.

The Optical–Pump–Terahertz–Probe (OPTP) spectroscopy system used in this project is schematically illustrated in **Figure S1**. The laser source is a Ti:sapphire regenerative amplifier that produces pulses with a central wavelength of 800 nm (1.55 eV) and a pulse duration of approximately 50 fs. The 800 nm output is split into three beams. The transmitted portion (90%) serves as the pump beam for the samples. This pump beam can be frequency-doubled to 3.1 eV using a BBO crystal or converted into the infrared or ultraviolet range using an optical parametric amplifier (OPA). The reflected portion (10%) of the laser is further divided into a generation beam and a sampling beam. The generation beam is directed through a 1-mm-thick ZnTe crystal to produce THz pulses, which are used to probe the sample. The pump and probe pulses are synchronized with a controllable delay (τ) and illuminate the sample with spot diameters of approximately 10 mm (pump) and ~1 mm (probe), respectively.

The OPTP system uses two independent delay stages: one for the pump and the other for the sampling beam. Experimental data were collected using both one-dimensional (e.g. with a fixed sampling delay and moving the pump delay) and two-dimensional scanning (moving both pump and sampling delay simultaneously) methods. In one-dimensional scanning, the sampling delay stage is fixed at either the peak of the THz pulse (for tracking the real conductivity dynamics) or the 0 crossing point (for measuring the imaginary conductivity), while the pump delay stage is varied to measure absorption of phase change in the THz pulse. In two-dimensional scanning, we sample out the probing terahertz pulse by simultaneously scanning the pump and sampling stages at a fixed pump-probe delay. By doing so, we ensure that different parts of the terahertz pulse can probe the same transient photoconductivity -this is particularly important for dynamics evolved much faster than the THz pulse duration.

1.1.2 Transmission electron microscopy (TEM)

TEM samples were prepared by drop-casting the hexane dispersion onto a carbon-coated copper grid. Routine TEM imaging was done using JEOL1400 TEM with an acceleration voltage of 120 kV.

1.1.3 Ultraviolet-visible (UV-vis) and photoluminescence (PL) spectroscopy

For optical spectroscopy, nanoplatelet dispersions were transferred to a quartz glass cuvette (1cm). UV-vis absorption spectra were recorded on a Cary 60 spectrophotometer. PL spectroscopy was recorded using a Prizmatix Silver high-power LED (emission peak 369 nm) for excitation and an Avantes SensLine AvaSpec-HSC-TEC for data collection.

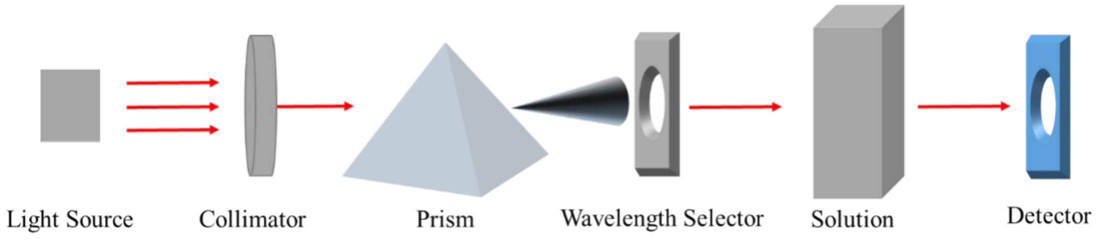

Figure S2 Schematic diagram of the UV-Vis spectrophotometer.

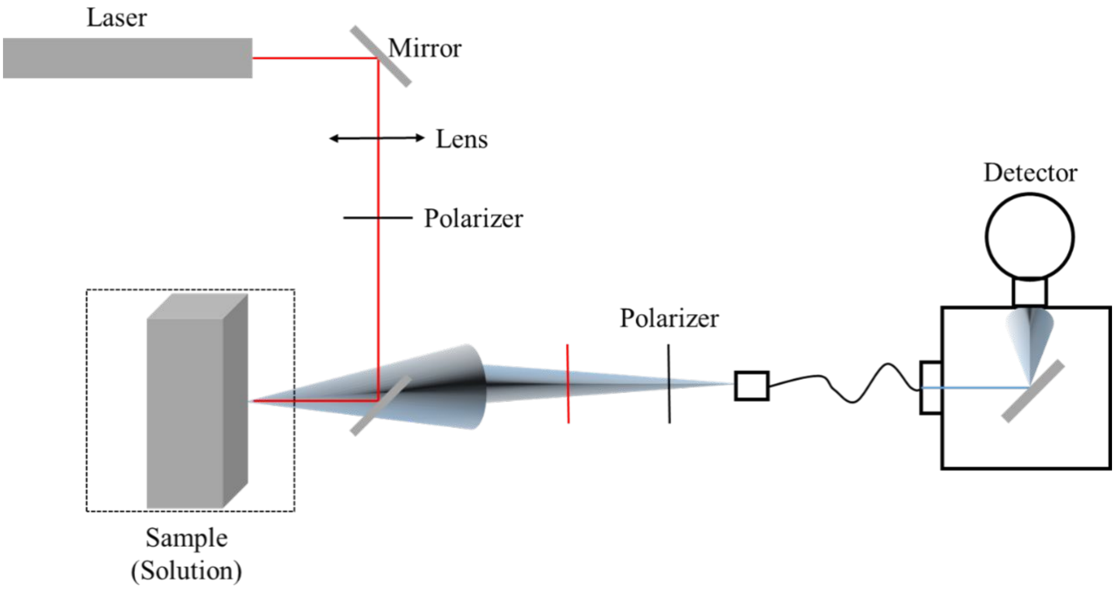

Figure S3 Schematic diagram of the photoluminescence setup.

1.1.4 Chemicals

All reagents were purchased and used without further purification. 1-Octadecene (ODE, Technical Grade, 90 %, 1 L), Oleic acid (OA, Technical Grade, 90 %, Sigma-Aldrich, 1 L), Myristic acid (> 98%, Sigma-Aldrich, 500 g), Cadmium acetate dihydrate ($Cd(OAc)_2.2H_2O$, 98%, Acros Organics, 50 g), Se (99.999 %, Alfa Aesar, 50 g), CdO (99%, Fisher Scientific, 250 g), Trifluoroacetic anhydride (TFAA, TCI, Tokoyo Chemical Industry, 100 mL), Trifluoroacetic acid (99.5%, TFA, Tokoyo Chemical Industry, 100 mL), Triethylamine (99.5%, TEA, Alfa Aesar, 1 L), Methanol (MeOH, HPLC Grade,

VWR Chemicals, 5 L). N-hexane (≥ 95%, Analytical Reagent Grade, 5 L) and Acetonitrile (CH_3CN, HPLC grade, 2.5 L) were purchased from Fisher Scientific Chemical. Methyl acetate (99%, HPLC grade, 2.5 L) was purchased from Merck KGaA.

1.1.5 Synthesis of cadmium myristate [Cd(myristate)$_2$].

Cadmium myristate (Cd(myr)$_2$) was synthesized following the procedure described by Rossinelli et al. In a typical synthesis of a 100 mL three-necked flask, 5.75 g (44.78 mmol) of CdO and 20 mL of acetonitrile were mixed and stirred at room temperature (RT, 500 rpm). And then, 0.7 mL (9.15 mmol) of trifluoroacetic acid (TFA) and 6.2 mL (43.98 mmol) of trifluoroacetic anhydride (TFAA) were slowly added to the mixture. Then, the mixture was stirred for an additional 10 min at RT. Afterward, the reaction mixture was heated up to 50 °C under stirring for 60 min. In another 500 mL beaker, 100 mL of 2-propanol, 14 mL (100.44 mmol) of triethylamine (TEA), and 10.23 g (44.80 mmol) of myristic acid were mixed. Then the cadmium trifluoroacetate solution was added slowly to the 500 mL baker while stirring (500 rpm). The product-white precipitate was vacuum-filtered and washed several (more than 4) times with 50 mL of cold methanol. Finally, the final product was collected, dried in a vacuum oven at 40 °C overnight, and stored under ambient conditions for further use.

1.1.6 Synthesis of 4 monolayer (4 ML) CdSe nanoplatelets

The 4 ML CdSe nanoplatelets were prepared by modifying the procedure published by Rebecca et al.[1] In a typical synthesis of a 100 mL three-necked flask, 170 mg (0.3 mmol) of Cd(myr)$_2$, 12 mg (0.15 mmol) of Se, and 15 mL of ODE were mixed. Then the mixture was degassed under a vacuum, heated up to 100 °C in 10 min, and refluxed at 100 °C for 13 min. Then, we turn off the vacuum and turn on the argon. Based on the inert atmosphere, the solution was heated up to 240 °C within 16 min. 80 mg (0.3 mmol) of Cd(ac)$_2$.2H_2O was added quickly to the mixture at 188 °C. After 6 min at 240 °C, the reaction mixture was quickly cooled down by a water bath. During the cooling step, 0.5 mL of OA was added at 185 °C. Finally, 5 mL of hexane was added to the mixture and centrifuged at 5000 rpm for 5 min. The supernatant was discarded, and the precipitate was dispersed in 5 mL hexane and centrifuged at 8000 rpm for another 5 min. The supernatant (4 ML+3 ML) was collected and stored. After one week, the remaining 3 ML nanoplatelets precipitated could be removed by centrifugation at 8000 rpm for 5 min. The 4 ML nanoplatelets in hexane were precipitated by using methyl acetate in a 1:1 volume ratio to remove residual ODE and oleic acid. Finally, the mixture was stored in the fridge at 5 °C for 1 h followed by another centrifugation at 8000 rpm for 10 min. The precipitate was dispersed in hexane. The dispersed nanoplatelets could be stored in hexane for several months.

By tuning the reaction time to 5-8 min at 240 °C, one can readily control the lateral area of the nanoplatelets from 24 to 420 nm^2.

1.1.7 Concentration-dependent PL emission measurements

To validate the OPTP method, we compare our results for the saturation concentrations with the alternative standard method of measuring the concentration-dependent PL emission, the relation of emitted to absorbed photons.[2] In the diluted limit, the PL efficiency normalized to the concentration (η_{PL}/c) is expected to show no dependence on the concentration. In other words, the PL efficiency increases linearly with concentration. However, we find that at a point η_{PL}/c goes down (**Figure S8**).

1.2 Additional experimental results

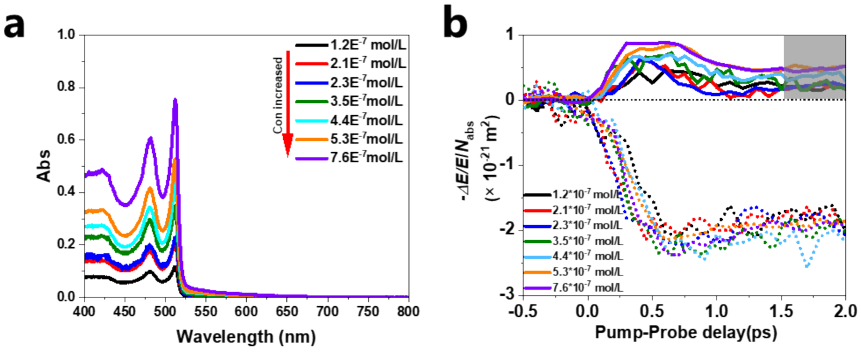

Figure S4 The UV-vis absorption spectra of NPLs-119 nm^2 (a) with different concentrations in hexane. The concentration-dependent, time-resolved THz conductivity following 3.1eV excitation of NPLs-119 nm^2 (b) in hexane.

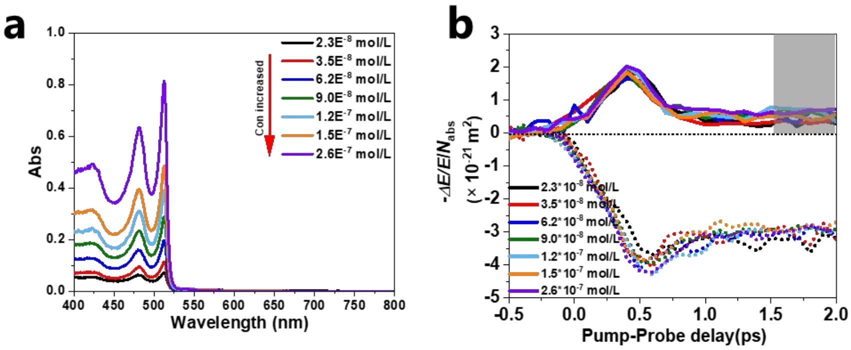

Figure S5 The UV-vis absorption spectra of NPLs-215 nm^2 (a) with different concentrations in hexane. The concentration-dependent, time-resolved THz conductivity following 3.1eV excitation of NPLs-215 nm^2 (b) in hexane.

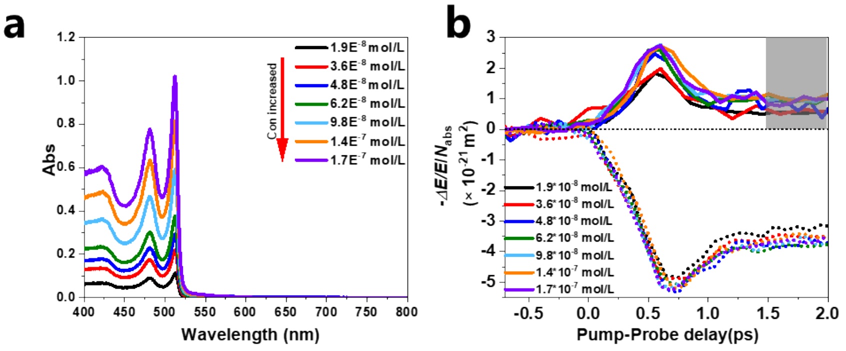

Figure S6 The UV-vis absorption spectra of NPLs-420 nm^2 (a) with different concentrations in hexane. The concentration-dependent, time-resolved THz conductivity following 3.1eV excitation of NPLs-420 nm^2 (b) in hexane.

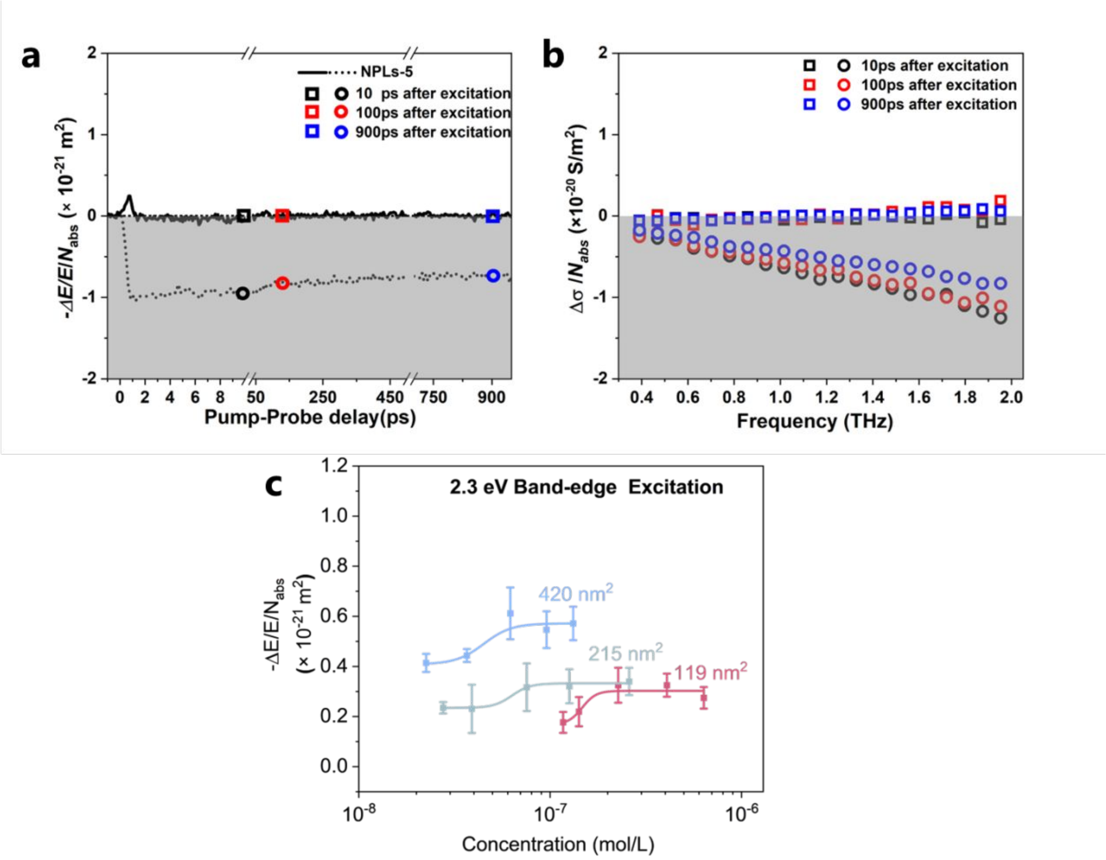

Figure S7 (a, b)The time-resolved THz conductivity following 3.1 eV excitation for NPLs-24 nm^2. (c) Concentration-dependent photoconductivity averaged between 1.5 ps to 2 ps of the real part of the photoconductivity normalized to the absorbed photon density following 2.3 eV excitation for NPLs-420 nm^2, 215 nm^2, and 119 nm^2.

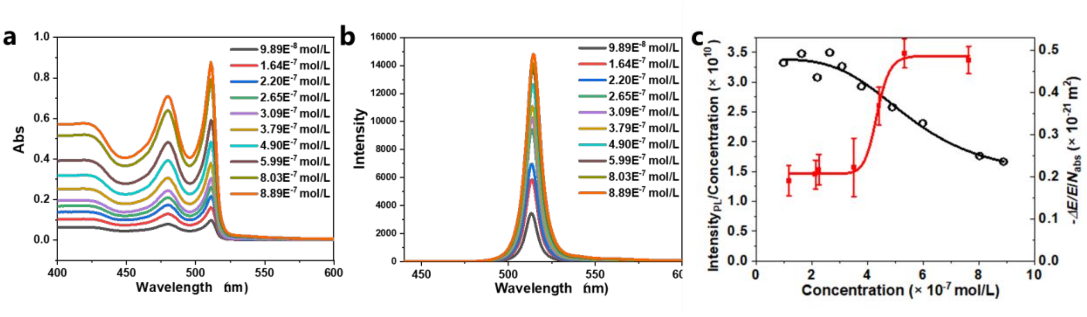

Figure S8 UV-vis absorption (a) and PL (b) spectra of NPLs-119 nm^2 with different concentrations. (c) Compare η_{PL}/c and OPTP measurements.

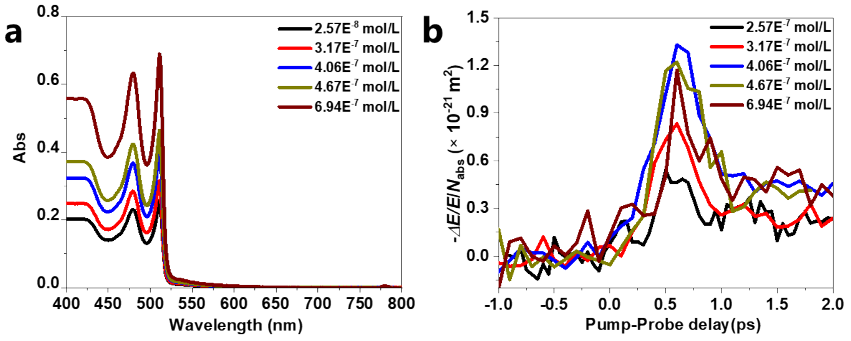

Figure S9 UV-vis absorption spectra of NPLs-119 nm^2 in octane (a) with different concentrations. The concentration-dependent, time-resolved THz conductivity following 3.1eV excitation of nanoplatelets in octane (b).

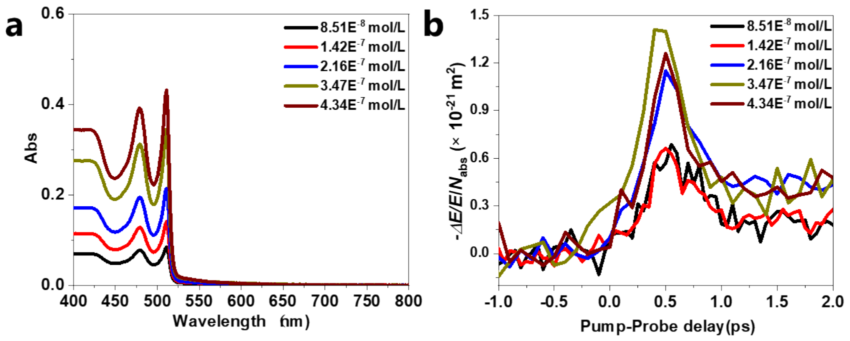

Figure S10 UV-vis absorption spectra of NPLs-119 nm^2 in decane (a) with different concentrations. The concentration-dependent, time-resolved THz conductivity following 3.1eV excitation of nanoplatelets in decane (b).

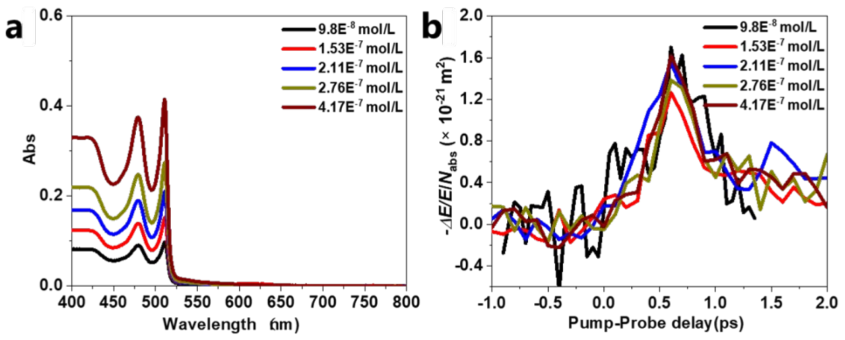

Figure S11 UV-vis absorption spectra of NPLs-119 nm^2 in dodecane (a) with different concentrations. The concentration-dependent, time-resolved THz conductivity following 3.1eV excitation of nanoplatelets in dodecane (b).

2 Simulations

We run molecular dynamics simulations with two similar setups, with different resolutions. The first setup is shown in **Figure S12a** and in **Figure 3b** in the main text. We model the nanocrystals via surface beads, which we in turn use as ligand grafting points. We do not include the core-core van der Waals interaction, or a potential present dipole-dipole interaction, since we expect them to be small.[3,4] However, we explicitly describe the ligand and solvent molecules, as well as their interaction. We have to describe a large number of beads in our simulations. Therefore, it is convenient to apply coarse graining here. In our first setup, we use the generic MARTINI force field to describe the ligand and solvent molecules.[5] This force field includes a four-to-one mapping. Four CH_x groups of an alkyl chain are mapped to one C1 Martini bead.

We use a chain of four C1 MARTINI beads to describe the ligands, and a ligand grafting density of 3.6 ligands per nm^2. This value is smaller than the experimental value of 5.4 ligands per nm^2 because of the coarse-graining. A more detailed description of the setup can be found in **Section 2.1.1** and our previous work.[3]

The second setup is very similar (**Figure S12b**). However, in this setup, we use the chemically accurate TraPPE-UA force field to describe the ligand and solvent molecules, as well as their interaction.[6] In this force field, each CH_x group is described by one bead. Therefore, we can describe the ligand and solvent molecules in a higher resolution, and can use here the experimental ligand grafting density of 5.4 ligands per nm^2 (see snapshots in **Figure 4a**). However, the higher resolution comes at the cost of reduced simulation speed. Therefore, we use this setup mainly in the second part of our simulations, where we need more details.

We begin our considerations with the comparison of different n-alkane solvents in the MARTINI setup. We consider solvent molecules with a length between one and four C1 MARTINI beads. This covers the range of n-alkanes that are liquid at room temperature (n-pentane to n-hexadecane).

Solvation forces are directly related to the layering of the solvent around the nanoplatelets. Therefore, we consider single nanoplatelets in the four n-alkane solvents and average the densities away from the base facets, see depiction in the inset of **Figure 3c**. We find no solvent-dependent change in the ligand densities (see **Figure S18**). However, the solvent densities show some notable features (**Figure 3c**).

In general, we find that the solvent density oscillates close to the ligand-solvent interface. The amplitudes of this oscillation increase with the n-alkane length. However, the spacing between the extrema does not change. This is because linear n-alkane molecules can align fully stretched parallel to the interface. Therefore, the spacing between the extremes in n-alkanes is always the same, regardless of the n-alkane length.[7] Also, the bulk solvent densities change. We find that the bulk solvent densities increase with the n-alkane length.

For comparison, we also calculate the solvent densities away from an infinite facet with the chemically accurate TraPPE-UA force field, see **Figure S19**. Here, we find similar trends.

In the next step, we consider the pair interaction between the base facets of two nanoplatelets. We use multiple window umbrella sampling in combination with the weighted histogram analysis method (WHAM) to calculate free energy curves (see setup in the inset of **Figure 3d**).[8,9,10,11,12] A detailed description can be found in **Section 2.1.4**. We find that the computational cost for the simulations increases drastically with the solvent length (see **Section 2.1.5**). Therefore, we consider in the simulations with four solvent beads only separations up to 6.3 nm distance.

We show the calculated free energy curves in **Figure 3d**. We place the zero point of the free energy in the 1st/global free energy minimum, since we have not calculated the full curve for three and four solvent beads. The convergence of the Umbrella-calculations decreases with the solvent length. We converged the free energy curves for solvent molecules with 1 and 2 MARTINI beads up to 9 nm surface-surface distance, while we converged the curve for 3 and 4 beads up to 4.8 nm surface-surface distance. We show the full curves in **Figure S20.**

As expected, we find that the free energy curve oscillates between attraction and repulsion due to the solvent layering between the nanoplatelets.[3] Overall, the curves have a similar shape. However, we find that the amplitudes of the free energy oscillation increase with the n-alkane length.

We find a relationship between the free energy curves and the ligand and solvent densities. The increase in the free energy amplitudes is in accordance with the increase of the amplitudes of the density oscillations around the nanoplatelets, and the increase in the bulk solvent density (**Figure 3c**). We assume that the larger changes in the densities and in the arrangements of the solvent molecules lead to an increase in the free energy differences between the different states.

This relation between the free energy and the density also becomes clear when comparing the densities at the 2nd and 1st free energy minimum (see **Figures S21** and **S22**). Again, we find that the ligand densities in the four solvents are nearly identical. However, the changes in the solvent densities become more pronounced with the n-alkane length. Accordingly, the free energy difference increases.

In **Figure 3d**, we also find that the extrema of the free energy curves slightly shift to smaller separations with increasing chain length. This can relate to a decrease in solvent density between the nanoplatelets (**Figures S21** and **S22**). Furthermore, we find in the free energy curve with four solvent beads a new local minimum that appears at around 4.8 nm distance, and whose characteristics we already find in the other curves. We assign it to the reorganizing and interdigitation of the ligands, which becomes more pronounced due to the increased attraction, and solvent depletion.

These simulation results show clearly that the attraction between the nanoplatelets increases with the n-alkane solvent chain length, while the change in the overall shape is only minor. However, it is well known that solvation forces also depend on the shape of the solvent molecules.[13,14,15] Branched isomers can be used to tune the interaction. For example, Wang et al. have shown that solvation forces between flat, uncoated hard surfaces are stronger in n-decane than in 2,2-dimethyloctane because of structural changes in the configuration of the solvent molecules.[14]

In our next step, we consider isomers of octane to show how the interaction between two ligand-coated nanoplatelets can be manipulated by the branching of alkane molecules. We compare n-octane

with 2-methylheptane, 3,4-dimethylhexane, and 2,2,4-trimethylpentane. We need a higher resolution to describe these solvent molecules. Therefore, we use here the TraPPE-UA setup. Snapshots of the solvent molecules from our simulations are shown at the top of **Figure 4a**.

Again, we average the ligand and solvent densities away from the base facet of single nanoplatelets. Also here, the ligand density is independent of the solvent type (**Figure S23**). However, we find differences in the solvent density (**Figure 3b**). We find that the thickness of the solvent layers increases from n-octane to 2-methylheptane, and to 3,4-dimethylhexane. The layers in 3,4-dimethylhexane, and 2,2,4-trimethylpentane have a very similar thickness. We assign the changes in the thickness of the solvent layers to the cross-section and the arrangement of the solvent molecules.

The branching also influences the amplitudes of the solvent density oscillation. We find that the amplitudes decrease from n-octane to 2-methylheptane, and to 3,4-dimethylhexane. However, the amplitudes increase again for 2,2,4-trimethylpentane. We attribute this increase to the compactness of the molecule.

The changes in the densities are reflected in the interaction of the nanoplatelets. In **Figure 4c**, we present free energy curves that describe the base facet-to-base facet interaction. Simulations within the TraPPE-UA setup include many more particles. Because of the higher computational cost, we limit ourselves to the first part of the free energy curves. Therefore, we place the zero-point of the free energy again in the first free energy minimum.

The free energy curves show qualitatively a very similar shape as those we have calculated with the MARTINI setup (see **Figure 3d, Figure S20**). The results confirm our assessment that the MARTINI model is suitable here. However, the higher resolution of the TraPPE-UA setup allows us to distinguish the characteristics of the octane isomers.

It is noticeable that the first minimum is always at ~3.44 nm and the free energy curves are almost identical up to a distance of ~3.65 nm, regardless of the solvent (**Figure 4c**). We assign this to the ligand and solvent densities between the nanoplatelets. Regardless of the octane isomer, the ligand densities are nearly identical at the 1st free energy minimum (see **Figure S24**). Furthermore, the solvent density between the ligand shells is nearly zero. Therefore, there is no difference in the course of the free energy curves at short distances.

The free energy curves split near the 1st maximum (**Figure 4c**). Additionally, the peak of the 1st maximum shifts to larger distances with increasing branching of isomers, and the maximum free energy value increases. For both changes, we assign to the increasing cross-section of the isomers.

Essentially, we observe here an excluded volume respectively depletion effect. Assume a simplified model with two hard, flat facets, no ligands, and hard spherical solvent molecules (see **Figure S25**). As the two facets depart from one another, an excluded volume appears between the facets. A certain distance must be reached before the solvent molecules can enter. As the distance between the facets increases, the excluded volume increases, and the free energy increases accordingly.

Accordingly, the maximum free energy depends on the size of the solvent molecules. For larger solvent molecules, the maximum peak shifts to a larger separation, and the maximum free energy value

increases. This is a very simplified model that neglects the softness of the ligand shell, the flexibility of the solvent molecules, and the change in the interactions of the molecules. Nevertheless, the model illustrates why the free energy difference between the 1st minimum and the 1st maximum free increases.

We find that the octane isomer influences the position of the 2nd minimum, too. Its position moves to larger values from n-octane, to 2-methylheptane, and to 3,4-dimethylhexane. The positions for 3,4-dimethylhexane and 2,2,4-trimethylpentane are nearly the same. We assign this to the change in the thickness of the solvent layers (**Figure 4b** and **Figure S24**).

While there is a clear relationship between the spacing of the extrema in the densities and the free energy, there is not necessarily a simple relationship between the free energy values and the density curves. 3,4-dimethylhexane and 2,2,4-trimethylheptane have nearly identical ligand and solvent density curves at the 1st and 2nd minimum (**Figure S24**). However, the free energy difference between the 1st and 2nd free energy minimum changes. This demonstrates that the free energy depends not only on the densities, but also on the excluded volume effect, and the interaction between the ligand and solvent molecules, which in turn depends on the solvent type.

In summary, we find that the interaction between the nanoplatelets depends on the effective interaction between the ligand and solvent molecules, as well as their arrangement. Furthermore, our simulations show that the solvent type can be used to tune the solvation forces between CdSe nanoplatelets.[15]

2.1 Technical details

2.1.1 Model system setups

We use two very similar setups of finite nanoplatelets in our molecular dynamics simulations (see **Figure S12a, b**). We model the nanocrystals of the nanoplatelets via surface beads, which we in turn use as grafting points for the ligand molecules. To replicate the surface of CdSe nanoplatelets, we arrange the ligand grafting points in a face-centered grid.[16,17] We assume that the core-core van der Waals attraction and a possible present dipole-dipole interaction are small.[3,4] Therefore, we do not include them in our description. However, we explicitly describe the ligand and solvent molecules, as well as their interactions. In the first setup, we use the MARTINI force field (**Figure S12a**),[5] while we use the TraPPE-UA force field in the second setup (**Figure S12b**).[6]

The MARTINI force field is a generic coarse-grained force field.[5] It includes a four-to-one mapping. Here, four CH_x groups are mapped to one C1 MARTINI bead (see **Figure S12d**). There is no representation of CdSe within the MARTINI force field. Here, we chose the most repelling interaction of the interaction table to describe the interaction between the surface beads and the ligand/solvent beads (see **Table S1**). This choice is reasonable since the effects we are interested in rely on the ligand-solvent interface. The interaction with the surface beads has only a minor effect on the interface. The theoretical and experimental confirmed ligand grafting density for carboxylic ligands on the CdSe nanoplatelets is 5.4 ligands/nm².[17,18] However, we use 3.6 ligands/nm² in the MARTINI setup because of the coarse-graining, which leads to larger beads.[3] We have already used the MARTINI setup in our previous publication.[3] We refer to it for a more comprehensive introduction to the details and for parameter tests.

In the second setup, we use the chemically more accurate TraPPE-UA force field. In this united atom force field, each CH_x group is described by one bead (**Figure S12d**). The interactions between the beads are described via 12-6 Lennard-Jones potentials. We use some deviations from the standard TraPPE-UA. Firstly, the original force field includes a tail correction. However, we use HOOMD blue in version 2.6 for our simulations.[19,20,21] Since this version does not include a tail correction, we instead increase the cutoff to r_{cutoff} = 1.8 nm. Secondly, the interactions between the beads of a molecule that are separated by less than four bonds are considered to be zero in the TraPPE-UA force field. This includes the surface beads in our nanoplatelet setup, which causes the problem that a part of the ligand molecules can enter the inner part of the nanoplatelet. We add additional surface beads between the ligand grafting points to solve this technical issue. Thirdly, we couldn't find interaction parameters specifically designed for CdSe. Therefore, we chose interaction parameters which actually describe the interaction with CdSe (see **Table S2**).[22] Again, this choice is reasonable since the effects we are interested in rely on the ligand-solvent interface, and since the interaction with the surface beads have only a minor effect on the interface. Fourthly, the bond length is fixed in the original TraPPE-UA force field. Instead, we use a bond stretching term.[23] Finally, we note that we use the expected ligand grafting density of 5.4 ligands per nm^2 in the TraPPE-UA setup.[17,18]

	CdSe	
	ϵ in kJ / mol	σ in nm
C1	2.0	0.62

Table S1 Interaction parameter for the interaction between the surface beads and the ligand/solvent beads that are used in the MARTINI setup.

	CdSe	
	ϵ in kJ / mol	σ in nm
CH_3	0.599	0.354
CH_2	0.466	0.354
CH	0.466	0.354

Table S2 Interaction parameter for the interaction between the surface beads and the ligand/solvent beads that are used in the TraPPE-UA setup.

We use the HOOMD package (v2.6) to run our molecular dynamics simulations.[19,20,21] We run our simulations at 300 K and 1 atm, and use cubic periodic simulation boxes. In the simulations with the TraPPE-UA setup, we use a Dissipative Particle Dynamics (DPD) thermostat together with a Martyna-Tobias-Klein barostat.[24,25] Here, we choose time steps of 2 fs. In the simulations with the MARTINI setup, we use the standard HOOMD NPT integrator to control the temperature and the pressure. The standard HOOMD NPT integrator is based on the Martyna-Tobias-Klein equations of motion.[25,26,27] Here, we use time steps of 20 fs (x4 for effective time).[3,5]

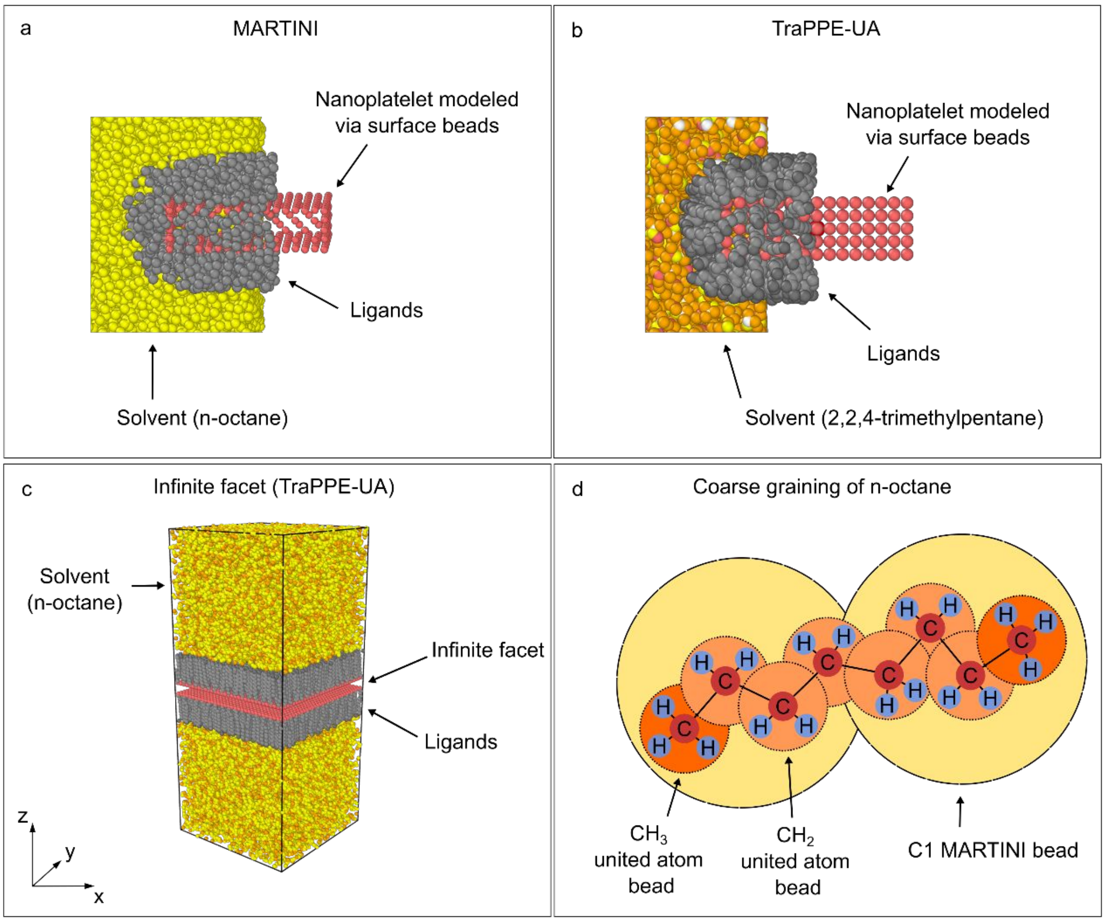

Figure S12 Model system setups for the molecular dynamics simulations. (a) and (b) show the setup of finite nanoplatelets. The nanocrystal surface is modeled via surface beads, which are used in turn as grafting points of the ligand molecules. (c) shows the setup of an double walled infinite facet. In the setups in (a) and (c), the TraPPE-UA force field is used to describe the ligand and solvent molecules, as well as their interactions. Here, additional surface beads between the ligand grafting points are used to prevent ligand molecules from entering the center of the nanoplatelets. In the setup in panel b, the MARINI force field is used. (d) shows a sketch of an n-octane molecule. The differences in the representation in the two force fields are shown.

2.1.2 Infinite facet setup

We create also a setup with two infinite facets. This setup is very similar to the TraPPE-UA setup for a finite nanoplatelet (see above). We create a double-walled facet, which we model via surface beads (see **Figure S12c**). In contrast to the nanoplatelet setup, we fix the position of the surface beads during the whole simulation. We use periodic simulation boxes, where we fix the x- and y-sides length. Thus the setup describes two infinite facets.

We use a Martyna-Tobias-Klein barostat to control the pressure via the z-side length of the simulation box,[25] together with a Dissipative Particle Dynamics (DPD) thermostat to control the temperature.[24] Again, we choose time steps of 2 fs.

2.1.3 Choice of the nanoplatelet dimensions and ligand length

We ran our simulations before we did the experiments. Therefore, we use slightly different nanoplatelet configurations in the simulations. In the experiments, the CdSe nanoplatelets have a thickness of 1.4 nm, while we use 1.2 nm in the TraPPE-UA setup and 1.5 nm in the MARTINI setup. In our previous publication, we discussed the effect of the nanoplatelet thickness on the MARTINI setup.[3] There, we have found that it has only a minor effect on the base facet-to-base facet interaction.

In our experiments, we use nanoplatelets with base facet areas between ~24 and ~420 nm^2. However, in our simulations, we are limited in the nanoplatelet size, which we can simulate. In the TraPPE-UA setup, we use a base facet area of 18.1 nm^2, while we use a base facet area of 35.6 nm^2 in the MARTINI setup.

In our previous publication, we discussed the effect of the base facet area.[3] There, we have found that the ligand packing density depends on the facet area. The ligands at the edges of the facets have more space, which they can occupy. Therefore, the overall ligand packing density is smaller on smaller facets, which influences the solvent layering away from the facets (see **Figure S15** and **Figure S16**). However, while the strength of the interaction scales with the facet area, the main features don't change.[3] Therefore, we assume that our results are transferable to larger nanoplatelets.

In our experiments, we use myristic ligands ($CH_3(CH_2)_{12}COOH$) with a chain length of 14 carbon atoms. In the MARTINI model, this translates to three or four C1 MARTINI beads. For the simulation in **Figure 3** of the main manuscript, we use four C1 beads to describe the ligands in the MARTINI setup, as we have done in our previous publication.[3] Since the strength of the interaction decreases with the ligand length, we probably underestimate the strength of the interaction.[3]

In contrast, we use ligands with a length corresponding to 8 carbon atoms in the simulations in **Figure 4** of the main manuscript. This represents octanoic acid ligands ($CH_3(CH_2)_6COOH$), which have been for example used by Jana *et al.*[28] We chose shorter ligands in this setup because of the small base facet area. Longer ligands would have a similar dimension as the base facets. Therefore, the edge effect would have a greater impact on the results. In **Figure S17**, we compare the densities away from the base facets of single nanoplatelets and infinite facets for different ligand lengths. With shorter ligands, the ligand packing density at the ligand-solvent interface is higher, and the solvent layering is more pronounced. In comparison with myristic ligands, the solvation forces per area will be stronger.[3]

2.1.4 Free energy calculation

We use multiple windows umbrella sampling to calculate free energy curves that describe the interaction of the CdSe nanoplatelets.[8] Thereby, we focus on the base facet-to-base facet interaction, since we assume that the solvation forces are most pronounced in this configuration (see sketch in **Figure S13**).[3] Thereby, we allow no rotation of the nanoplatelets, and only a movement of the nanoplatelets in the direction normal to the base facets.

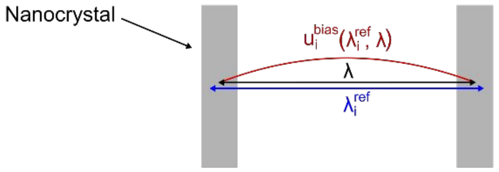

Figure S13 Sketch of the umbrella sampling setup.

In the simulations, we control the distance between the nanoplatelets via an artificial harmonic umbrella potential:

$$u_i^{\text{bias}}(\lambda_i^{\text{ref}},\lambda) = \frac{1}{2}\kappa(\lambda - \lambda_i^{\text{ref}})^2$$

where λ is the distance between the nanoplatelets, λ_i^{ref} the equilibrium distance of window i, and κ the force constant. Here, we use always $\kappa = 50000\ \text{kJ/ mol}$.

In **Figure S14**Figure S, we sketch our workflow. We first create an initial configuration. Then, we divide the reaction coordinate λ into windows i with λ_i^{ref}, and into intervals. The chosen difference $\Delta\lambda^{\text{ref}}$ depends on the simulation, see **Table S3** and **Table S4**. We start the first window of each interval from the initial configuration. The other windows of each interval are initiated and run sequentially. After the sequential run, we continue the simulations until they reach their equilibrium.

After the equilibration of the simulations, we collect the center-center distances in each window in a production run. In the simulations with the MARTINI setup, we use a production run with a length of 40 ns for one and two C1 solvent beads and a length of 80 ns for three and four C1 solvent beads (effective time[29]).[3,5] In the first case, we collect the distances from 250 snapshots, while we collect them from 500 snapshots in the second case. In the simulations with the TraPPE-UA setup, the production run has a length of 2 ns, where we collect the distances from 500 snapshots.

In the last step, we use the probability distributions of the center-center distances in each window to calculate the free energy curve with the Weighted Histogram Analysis Method (WHAM).[30,11,9,12] Here,

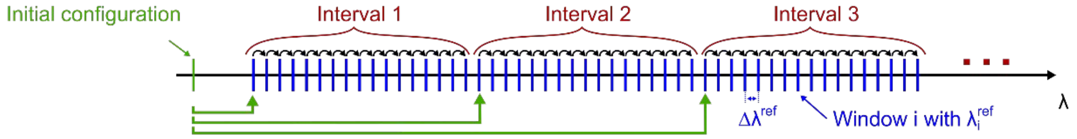

Figure S14 Sketch of the umbrella sampling workflow. The reaction coordinate $\boldsymbol{\lambda}$ is divided into windows **i** with $\boldsymbol{\lambda_i^{\text{ref}}}$, and into intervals. First an initial configuration is created. Then, the first window of each interval is started from the initial configuration. The other windows of each interval are initiated and run sequentially.

we use the tool of D. Bauer,[9] where we use 305 bins/nm, a tolerance 10^{-6} kJ/mol, and ten bootstrapping runs to calculate the error (see the SI of our previous publication for parameter tests).[3]

2.1.5 Simulation parameter

# of C1 MARTINI beads in each solvent molecule	Base facet area (nm^2)	# of solvent molecules	Average simulation box side length (nm)	$\Delta\lambda^{\mathrm{ref}}$ (nm)
1	35.6	200k	29.6	0.0025
2	35.6	250k	38.6	0.0025
3	35.6	450k	53.0	0.0025
4	35.6	500k	60.0	0.0025

Table S3 Simulation details for the analysis of different long n-alkane solvents on the interaction between CdSe nanoplatelets. (See **Figure 3** of the main manuscript, **Figure S20**, **Figure S21**, and **Figure S22**).

Solvent type	Base facet area (nm^2)	# of solvent molecules	Average simulation box side length (nm)	$\Delta\lambda^{\mathrm{ref}}$ (nm)
n-octane	18.1	100k	30.1	0.005
2-methylheptane	18.1	100k	30.2	0.005
3,4-dimethylhexane	18.1	100k	29.9	0.005
2,2,4-trimethylpentane	18.1	100k	30.3	0.005

Table S4 Simulation details for the analysis of different isomers of octane on the interaction between CdSe nanoplatelets (see **Figure 4** of the main manuscript, **Figure S23, and Figure S24**).

Base facet area (nm^2)	# of solvent molecules	x=y side length (nm)	Average simulation z-side length (nm)
2.2	100k		28.4
5.0	100k		28.4
8.9	100k		28.4
13.9	100k		28.5
20.0	100k		28.5
27.3	100k		28.5
35.6	250k		38.6
45.1	250k		38.6
55.7	350k		43.2
67.4	350k		43.2
80.2	350k		43.2
94.1	350k		43.2
245.5	350k		43.3
Infinite facet	5k	7.5	24.8

Table S5 Simulation details for the analysis of the facet area in the Martini setup (see **Figure S15**). For the finite nanoplatelets, the average simulation box side are x=y=z. In the case of the infinite facet, the x and y side lengths of the simulation box are kept fixed during the simulation.

Base facet area (nm^2)	# of solvent molecules	x=y side length (nm)	Average simulation z-side length (nm)
5.9	50k		24.0
9.2	50k		24.0
13.3	50k		24.0
18.1	50k		24.1
23.7	50k		24.1
29.9	50k		24.2
37.0	50k		24.2
Infinite facet	6k	9.7	24.4

Table S4 Simulation details for the analysis of the facet area in the TraPPE-UA setup (see **Figure S16**). For the finite nanoplatelets, the average simulation box side are x=y=z. In the case of the infinite facet, the x and y side lengths of the simulation box are kept fixed during the simulation.

Base facet area (nm^2)	# of ligand beads	# of solvent molecules	x=y side length (nm)	Average simulation z-side length (nm)
18.1	8	50k		34.3
18.1	12	50k		34.3
18.1	16	50k		34.4
18.1	20	50k		34.5
Infinite facet	8	6k	9.7	20.4
Infinite facet	12	6k	9.7	21.4
Infinite facet	16	6k	9.7	22.4
Infinite facet	20	6k	9.7	23.4

Table S5 Simulation details for the analysis of the ligand length in the TraPPE-UA setup (see **Figure S17**). For the finite nanoplatelets, the average simulation box side are x=y=z. In the case of the infinite facet, the x and y side lengths of the simulation box are kept fixed during the simulation.

Base facet area (nm^2)	Solvent type	# of solvent molecules	x=y side length (nm)	Average simulation z-side length (nm)
Infinite facet	n-hexane	2k	4.9	23.8
Infinite facet	n-heptane	1.5k	4.9	20.7
Infinite facet	n-octane	6k	9.7	22.4
Infinite facet	n-nonane	1.5k	4.9	24.1
Infinite facet	n-decane	1.5k	4.9	25.7
Infinite facet	n-undecane	1.5k	4.9	27.4
Infinite facet	n-dodecane	1.5k	4.9	29.1
Infinite facet	n-tridecane	1.5k	4.9	30.8
Infinite facet	n-tetradecane	1.5k	4.9	32.5

Table S6 Simulation details for the analysis of different n-alkane solvents in an infinite facet setup, with the TraPPE-UA force field (see **Figure S19**). The x and y side lengths of the simulation box are kept fixed during the simulation.

2.2 Additional simulation results

Here, we present some additional simulation results.

2.2.1 Facet area

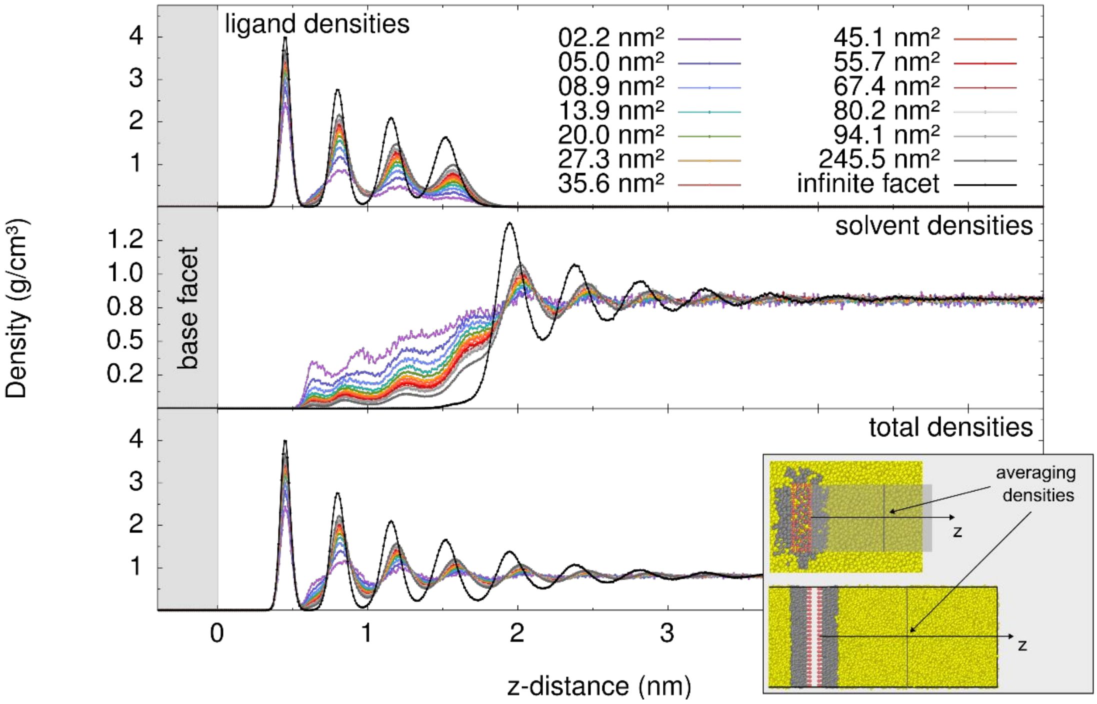

Figure S15 Comparison of the densities away from single nanoplatelets with different base facet areas in the MARTINI setup. The densities away from an infinite facet are also shown. The top panel shows the ligand, the middle panel the solvent, and the bottom panel the total densities. As depicted in the inset, the densities at each distance in z-direction are averaged over the whole facet area in the corresponding xy-plane. The solvent is n-octane. The solvent molecules are described by two C1 MARTINI beads. For clarity, the data points are connected via straight lines. Simulation parameters can be found in **Table S5**.

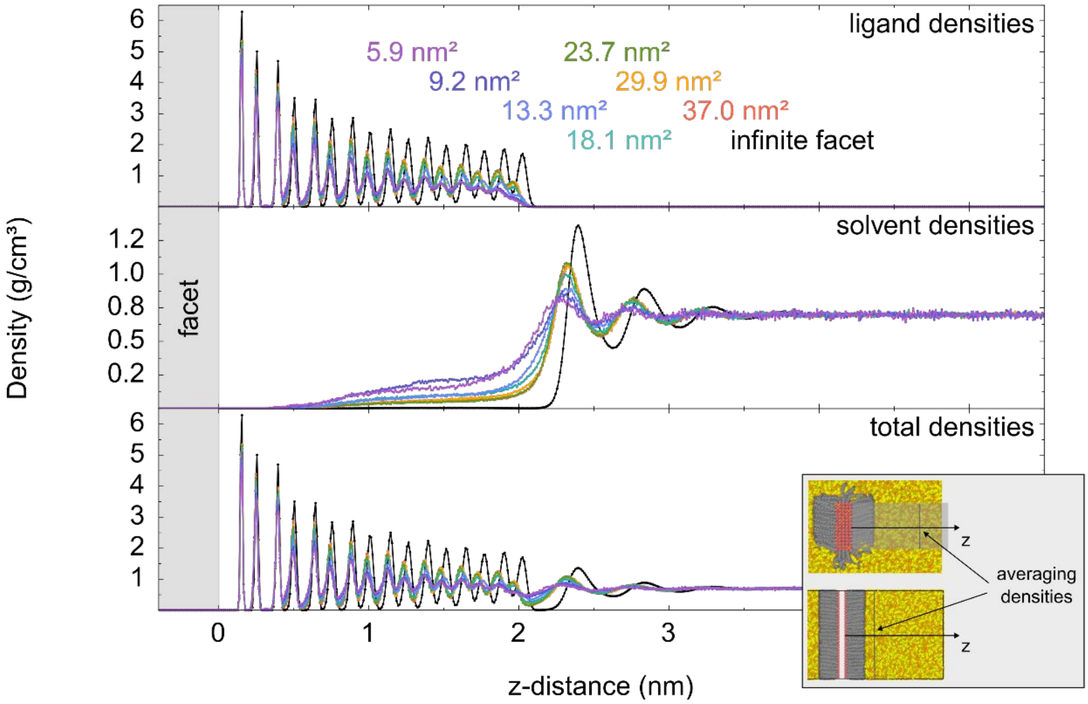

Figure S16 Comparison of the densities away from single nanoplatelets with different base facet areas in the TraPPE-UA setup. Here, the ligands have a length corresponding to 16 carbon atoms. The densities away from an infinite facet are also shown. The top panel shows the ligand, the middle panel the solvent, and the bottom panel the total densities. As depicted in the inset, the densities at each distance in *z*-direction are averaged over the whole facet area in the corresponding *xy*-plane. For clarity, the data points are connected via straight lines. Simulation parameters can be found in **Table S6**.

2.2.2 Ligand length

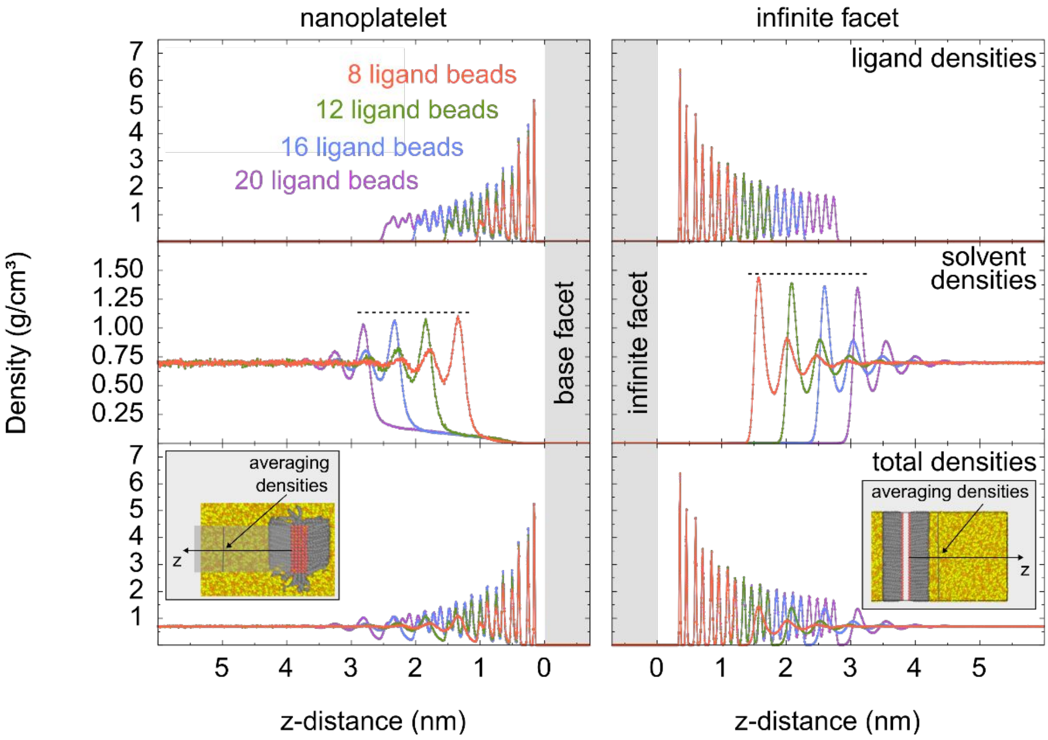

Figure S17 Comparison of different ligand lengths in the TraPPE-UA setup. The left column shows the densities away from the base facets of single nanoplatelets, while the right column shows the densities away from an infinite facet. The top panel shows the ligand, the middle panel the solvent, and the bottom panel the total densities. As depicted in the inset, the densities at each distance in the *z*-direction are averaged over the whole facet area in the corresponding *xy*-plane. For clarity, the data points are connected via straight lines. Simulation parameters can be found in **Table S7**.

2.2.3 n-alkane solvent length

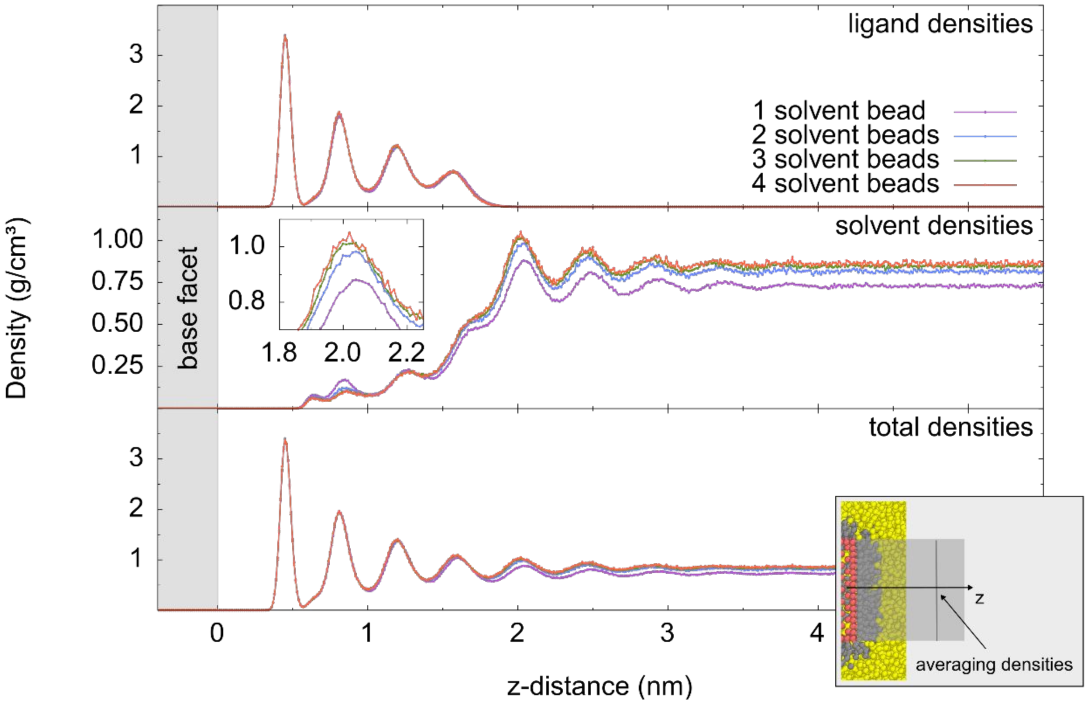

Figure S18 Effect of the n-alkane solvent length on the ligand and solvent density away from the base facet of a single nanoplatelet in the MARTINI setup. The top panel shows the ligand, the middle panel the solvent, and the bottom panel the total densities. The inset in the middle row shows a zoom of the largest maximum of the solvent densities. As depicted in the bottom row inset, the densities at each distance in *z*-direction are averaged over the whole facet area in the corresponding *xy*-plane. For clarity, the data points are connected via straight lines. Simulation parameters can be found in **Table S3**. Simulation parameters can be found in **Table S3.**

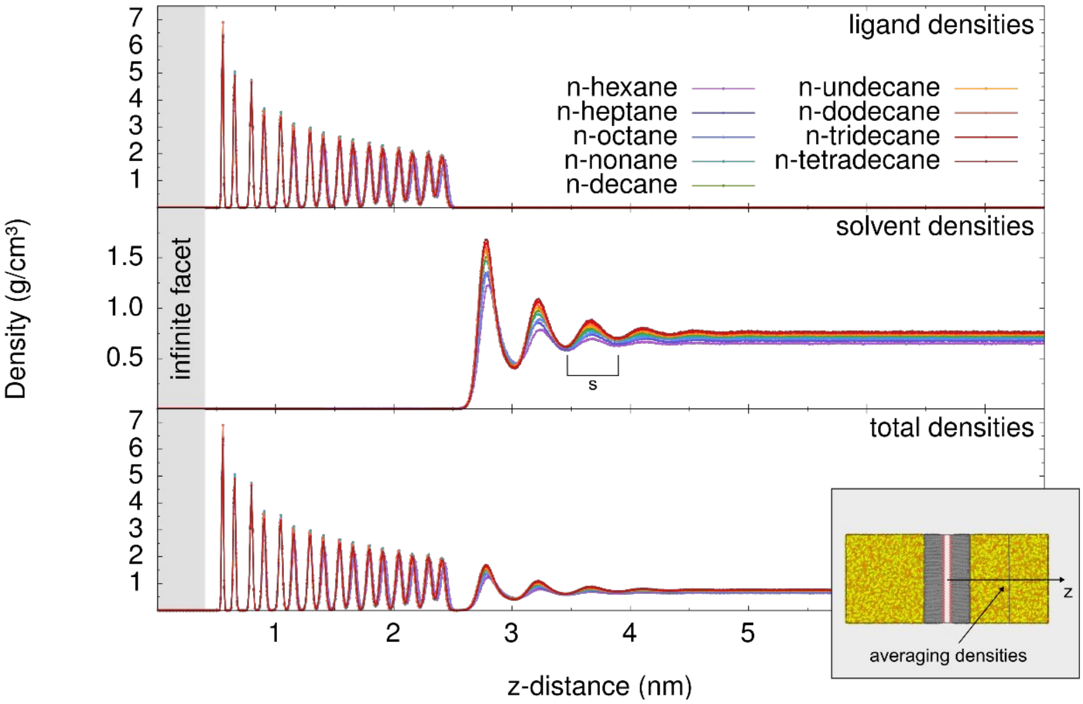

Figure S19 Effect of the n-alkane solvent length on the ligand and solvent density away from an infinite facet. The TraPPE-UA force field has been used to describe the ligand and solvent molecules and their interactions. Here the ligands have a length corresponding to 16 carbon atoms. The top panel shows the ligand, the middle panel the solvent, and the bottom panel the total densities. As depicted in the inset, the densities at each distance in *z*-direction are averaged over the facet area in the corresponding *xy*-plane. For clarity, the data points are connected via straight lines. Simulation parameters can be found in **Table S8**.

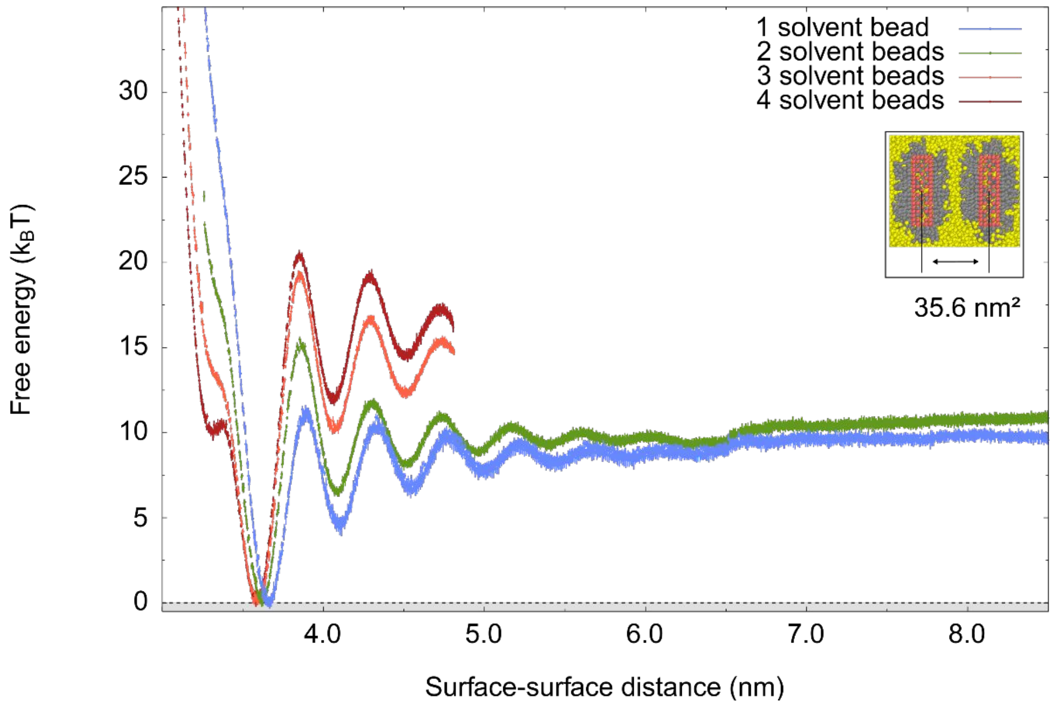

Figure S20 Comparison of n-alkane solvent length. Full converged free energy curves, see **Figure 3d** of the main manuscript. The convergence of the Umbrella-calculation decreases with the solvent length. We converged the free energy curves for solvent molecules with 1 and 2 MARTINI beads up to 9 nm surface-surface distance, while we converged the curves for 3 and 4 beads only up to 4.8 nm surface-surface distance. Simulation parameters can be found in **Table S3**.

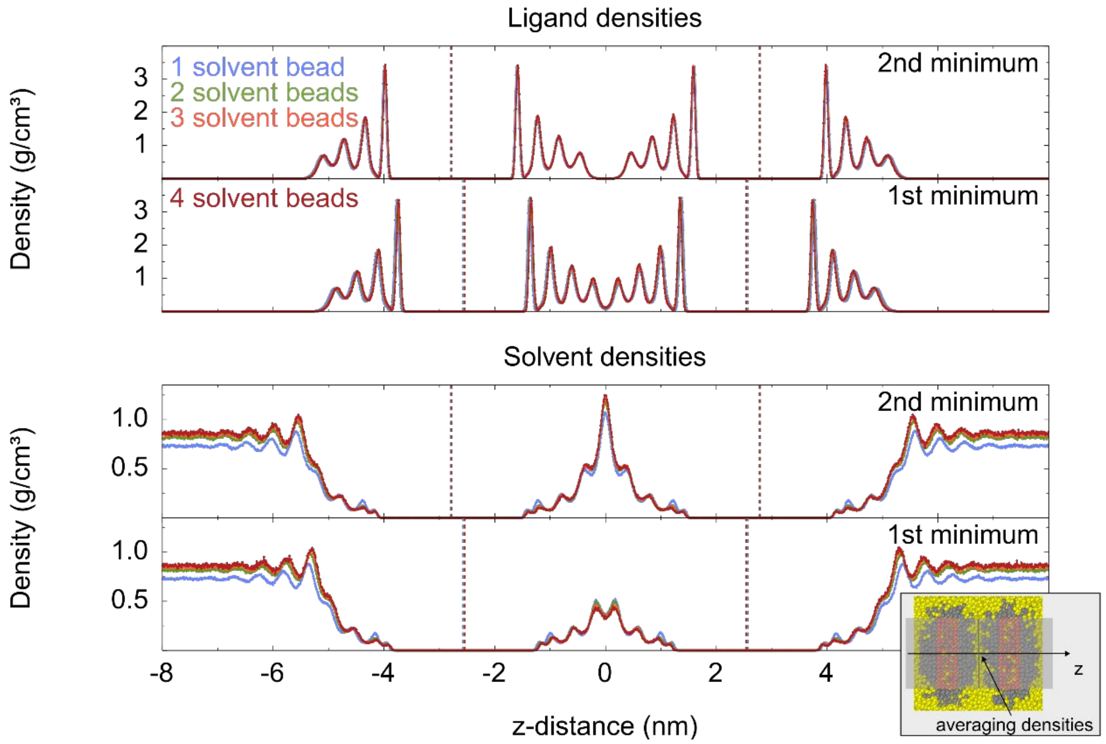

Figure S21 Comparison of n-alkane solvent length from the free energy calculation in **Figure 3d** of the main manuscript. Shown are the Ligand and solvent densities away and between the base facets in the first and second free energy minimum (see inset). A zoom of the solvent densities between the nanoplatelets is shown in **Figure S21**. Simulation parameters can be found in **Table S3**.

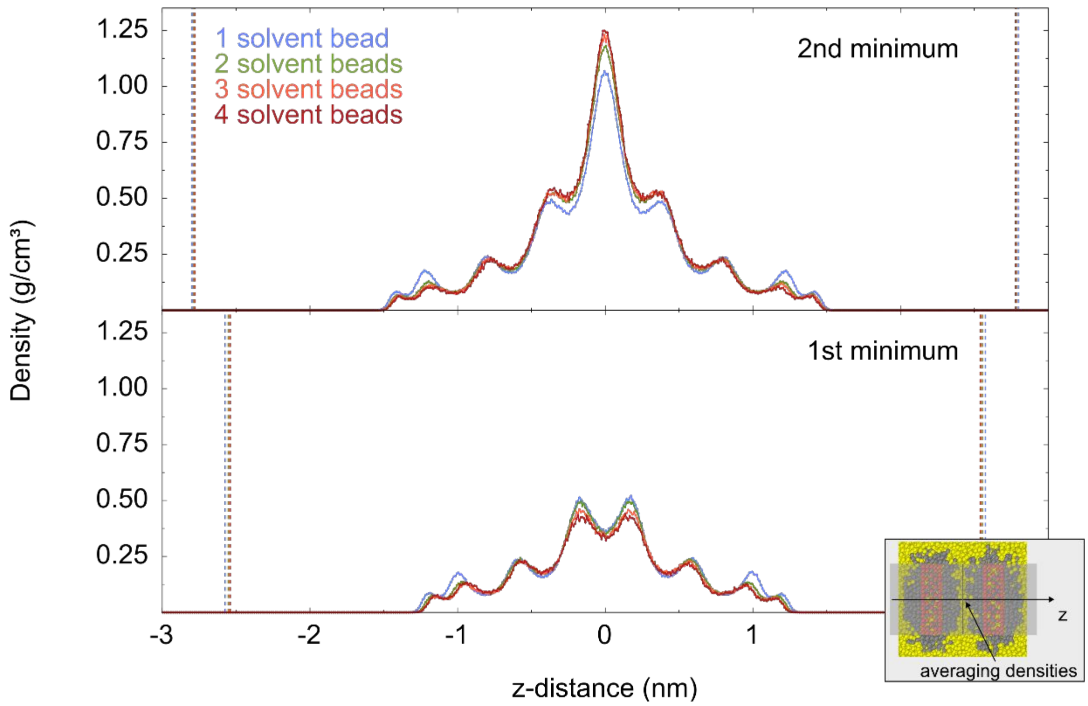

Figure S22 Zoom of the solvent densities between the nanoplatelets at the second and first free energy minimum. Simulation parameters can be found in **Table S3**.

2.2.4 Isomers of octane

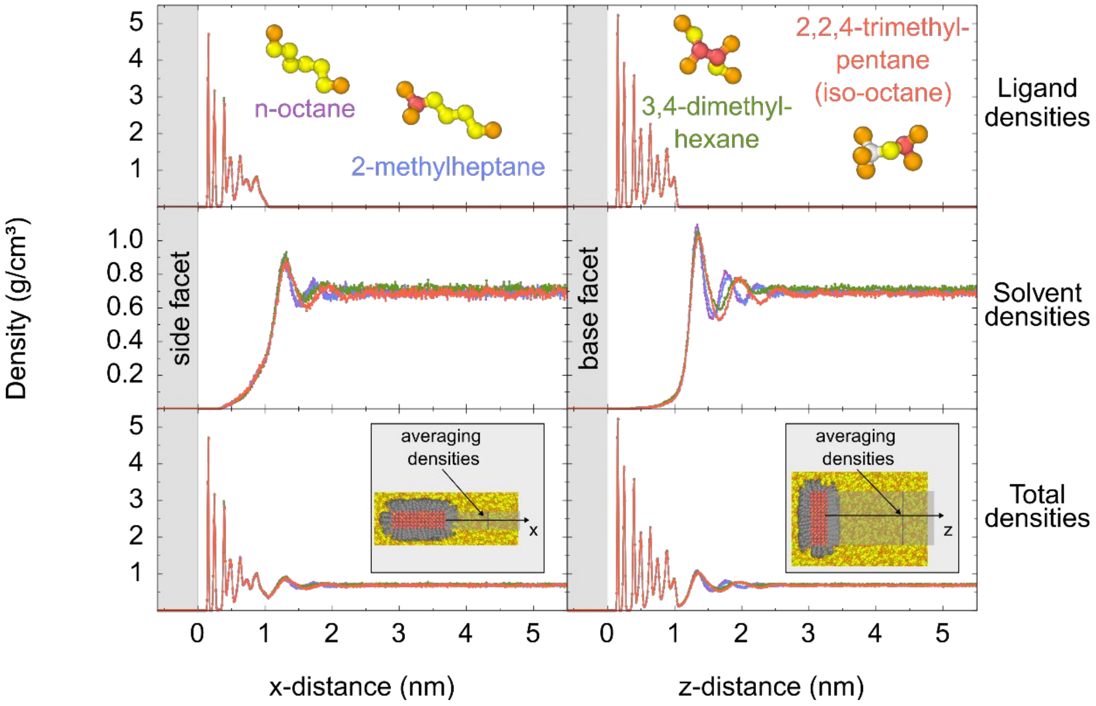

Figure S23 Comparison of different octane isomers. Shown are the averaged ligand and solvent densities away from a side facet of single nanoplatelets (left) and away from a base facet (right). The top panel shows the ligand, the middle panel the solvent, and the bottom panel the total densities. As depicted in the insets, the densities at each distance in the *x*/*z* direction are averaged over the whole facet area in the corresponding *zy*/*xy*-plane. For clarity, the data points are connected via straight lines. Simulation parameters can be found in **Table S4**.

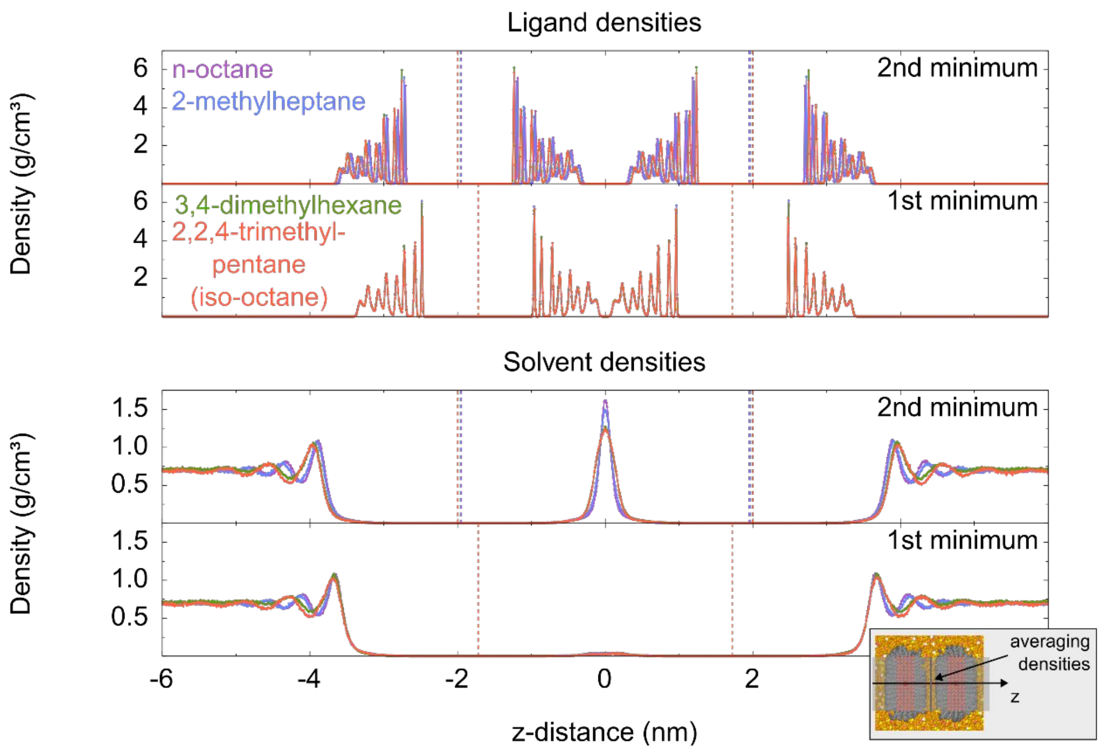

Figure S24 Comparison of different isomers of octane. Shown are the Ligand and solvent densities away and between the base facets in the first and second free energy minimum (see inset). Simulation parameters can be found in **Table S4**.

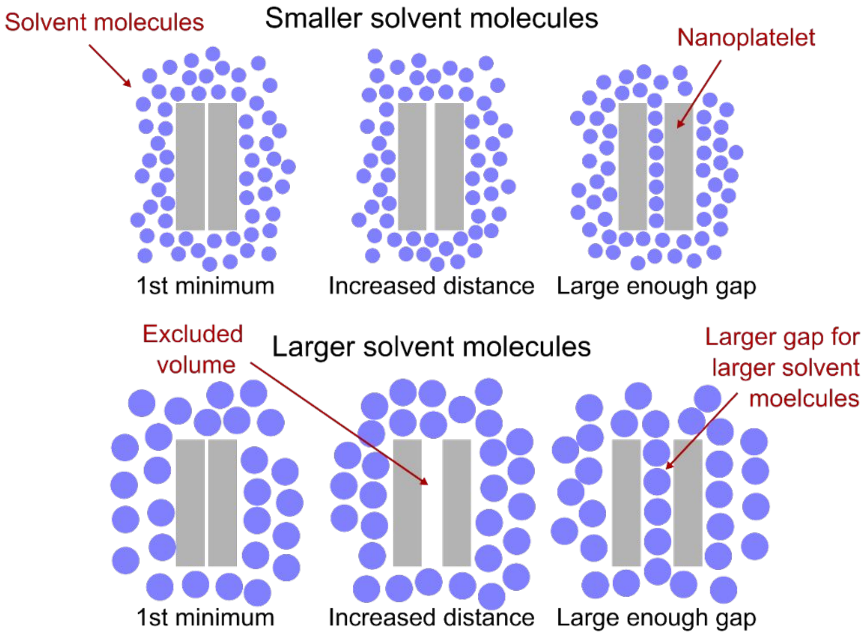

Figure S25 Simplified hard particle model to illustrate the excluded volume effect for solvent molecules with different sizes.

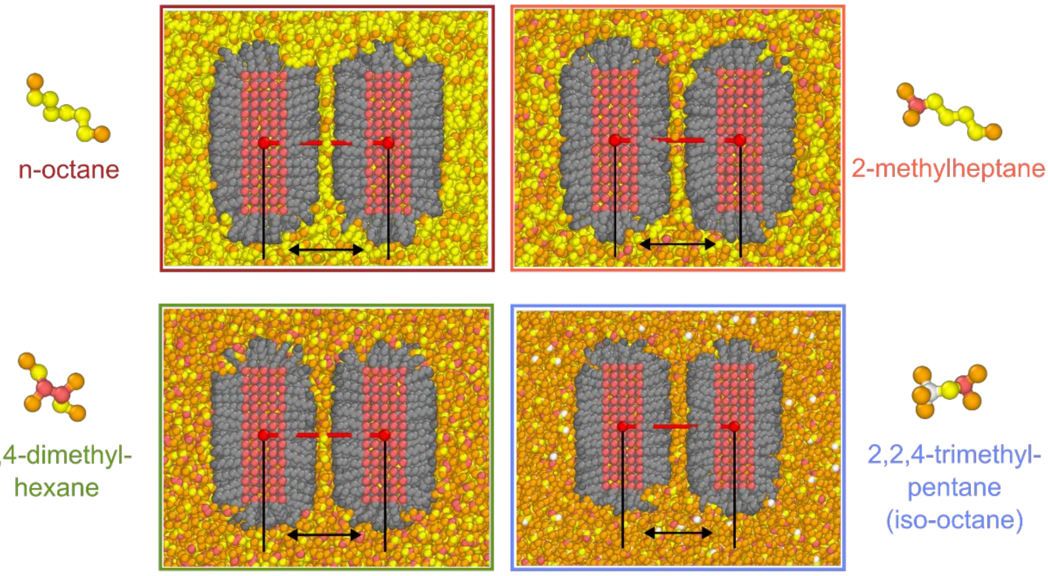

Figure S26 Snapshots from the calculations of the free energy curves close to the 2nd free energy minimum in **Figure 4c**.

References

(1) Momper, R.; Zhang, H.; Chen, S.; Halim, H.; Johannes, E.; Yordanov, S.; Braga, D.; Blülle, B.; Doblas, D.; Kraus, T.; et al. Kinetic Control over Self-Assembly of Semiconductor Nanoplatelets. *Nano Letters* **2020**, *20* (6), 4102–4110. DOI: 10.1021/acs.nanolett.9b05270.

(2) Halim, H.; Trieb, D.; Huber, N.; Martínez-Negro, M.; Meyer, L.-A.; Basché, T.; Morsbach, S.; Zhang, K. A. I.; Riedinger, A. Lateral Size Dependence in FRET between Semiconductor Nanoplatelets and Conjugated Fluorophores. *The Journal of Physical Chemistry C* **2020**, *124* (45), 25028–25037. DOI: 10.1021/acs.jpcc.0c06199.

(3) Petersen, N.; Girard, M.; Riedinger, A.; Valsson, O. The Crucial Role of Solvation Forces in the Steric Stabilization of Nanoplatelets. *Nano Letters* **2022**, *22* (24), 9847–9853. DOI: 10.1021/acs.nanolett.2c02848.

(4) Dozov, I.; Goldmann, C.; Davidson, P.; Abécassis, B. Probing permanent dipoles in CdSe nanoplatelets with transient electric birefringence. *Nanoscale* **2020**, *12* (20), 11040–11054, 10.1039/D0NR00884B. DOI: 10.1039/D0NR00884B.

(5) Marrink, S. J.; Risselada, H. J.; Yefimov, S.; Tieleman, D. P.; de Vries, A. H. The MARTINI Force Field: Coarse Grained Model for Biomolecular Simulations. *The Journal of Physical Chemistry B* **2007**, *111* (27), 7812–7824. DOI: 10.1021/jp071097f.

(6) Martin, M. G.; Siepmann, J. I. Transferable Potentials for Phase Equilibria. 1. United-Atom Description of n-Alkanes. *The Journal of Physical Chemistry B* **1998**, *102* (14), 2569–2577. DOI: 10.1021/jp972543.

(7) Christenson, H. K.; Gruen, D. W. R.; Horn, R. G.; Israelachvili, J. N. Structuring in Liquid Alkanes between Solid Surfaces: Force Measurements and Mean-Field Theory. *J. Chem. Phys.* **1987**, *87* (3), 1834–1841. DOI: 10.1063/1.453196.

(8) Kästner, J. Umbrella sampling. *WIREs Computational Molecular Science* **2011**, *1* (6), 932–942. DOI: https://doi.org/10.1002/wcms.66.

(9) *An efficient weighted histogram analysis implementation written in Rust. (v1.1.3) [Software].* 2021.
(10) Kumar, S.; Bouzida, D.; Swendsen, R. H.; Kollman, P. A.; Rosenberg, J. M. The weighted histogram analysis method for free-energy calculations on biomolecules. I: The method. *J. Comput. Chem.* **1992**, *13* (8), 1011–1021. DOI: 10.1002/jcc.540130812.
(11) Souaille, M.; Roux, B. t. Extension to the weighted histogram analysis method: combining umbrella sampling with free energy calculations. *Computer Physics Communications* **2001**, *135* (1), 40–57. DOI: https://doi.org/10.1016/S0010-4655(00)00215-0.
(12) Hub, J. S.; de Groot, B. L.; van der Spoel, D. g_wham—A Free Weighted Histogram Analysis Implementation Including Robust Error and Autocorrelation Estimates. *Journal of Chemical Theory and Computation* **2010**, *6* (12), 3713–3720. DOI: 10.1021/ct100494z.
(13) Christenson, H. K. Experimental measurements of solvation forces in nonpolar liquids. *The Journal of Chemical Physics* **1983**, *78* (11), 6906–6913. DOI: 10.1063/1.444639.
(14) Wang, J.-C.; Fichthorn, K. A. Molecular dynamics studies of the effects of chain branching on the properties of confined alkanes. *The Journal of Chemical Physics* **2002**, *116* (1), 410–417. DOI: 10.1063/1.1419258.
(15) Preface to the Third Edition. In *Intermolecular and Surface Forces (Third Edition)*, Israelachvili, J. N. Ed.; Academic Press, 2011; p xvii.
(16) Ko, J.-H.; Yoo, D.; Kim, Y.-H. Atomic models for anionic ligand passivation of cation-rich surfaces of IV–VI, II–VI, and III–V colloidal quantum dots. *Chemical communications* **2017**, *53* (2), 388–391, 10.1039/C6CC07933D. DOI: 10.1039/C6CC07933D.
(17) Zhang, J.; Zhang, H.; Cao, W.; Pang, Z.; Li, J.; Shu, Y.; Zhu, C.; Kong, X.; Wang, L.; Peng, X. Identification of Facet-Dependent Coordination Structures of Carboxylate Ligands on CdSe Nanocrystals. *Journal of the American Chemical Society* **2019**, *141* (39), 15675–15683. DOI: 10.1021/jacs.9b07836.
(18) Guillemeney, L.; Lermusiaux, L.; Landaburu, G.; Wagnon, B.; Abécassis, B. Curvature and self-assembly of semi-conducting nanoplatelets. *Communications Chemistry* **2022**, *5* (1), 7. DOI: 10.1038/s42004-021-00621-z.
(19) Anderson, J. A.; Glaser, J.; Glotzer, S. C. HOOMD-blue: A Python package for high-performance molecular dynamics and hard particle Monte Carlo simulations. *Computational Materials Science* **2020**, *173*, 109363. DOI: https://doi.org/10.1016/j.commatsci.2019.109363.
(20) Anderson, J. A.; Lorenz, C. D.; Travesset, A. General purpose molecular dynamics simulations fully implemented on graphics processing units. *Journal of Computational Physics* **2008**, *227* (10), 5342–5359. DOI: https://doi.org/10.1016/j.jcp.2008.01.047.
(21) Glaser, J.; Nguyen, T. D.; Anderson, J. A.; Lui, P.; Spiga, F.; Millan, J. A.; Morse, D. C.; Glotzer, S. C. Strong scaling of general-purpose molecular dynamics simulations on GPUs. *Computer Physics Communications* **2015**, *192*, 97–107. DOI: https://doi.org/10.1016/j.cpc.2015.02.028.
(22) Widmer-Cooper, A.; Geissler, P. Orientational Ordering of Passivating Ligands on CdS Nanorods in Solution Generates Strong Rod–Rod Interactions. *Nano Letters* **2014**, *14* (1), 57–65. DOI: 10.1021/nl403067p.
(23) Nath, S. K.; Escobedo, F. A.; de Pablo, J. J. On the simulation of vapor–liquid equilibria for alkanes. *The Journal of Chemical Physics* **1998**, *108* (23), 9905–9911. DOI: 10.1063/1.476429.
(24) Phillips, C. L.; Anderson, J. A.; Glotzer, S. C. Pseudo-random number generation for Brownian Dynamics and Dissipative Particle Dynamics simulations on GPU devices. *Journal of Computational Physics* **2011**, *230* (19), 7191–7201. DOI: https://doi.org/10.1016/j.jcp.2011.05.021.

(25) Martyna, G. J.; Tobias, D. J.; Klein, M. L. Constant pressure molecular dynamics algorithms. *The Journal of Chemical Physics* **1994**, *101* (5), 4177-4189. DOI: 10.1063/1.467468 (acccessed 8/21/2024).
(26) Yu, T.-Q.; Alejandre, J.; López-Rendón, R.; Martyna, G. J.; Tuckerman, M. E. Measure-preserving integrators for molecular dynamics in the isothermal–isobaric ensemble derived from the Liouville operator. *Chemical Physics* **2010**, *370* (1), 294–305. DOI: https://doi.org/10.1016/j.chemphys.2010.02.014.
(27) Tuckerman, M. E.; Alejandre, J.; López-Rendón, R.; Jochim, A. L.; Martyna, G. J. A Liouville-operator derived measure-preserving integrator for molecular dynamics simulations in the isothermal–isobaric ensemble. *Journal of Physics A: Mathematical and General* **2006**, *39* (19), 5629. DOI: 10.1088/0305-4470/39/19/S18.
(28) Jana, S.; Phan, T. N. T.; Bouet, C.; Tessier, M. D.; Davidson, P.; Dubertret, B.; Abécassis, B. Stacking and Colloidal Stability of CdSe Nanoplatelets. *Langmuir* **2015**, *31* (38), 10532–10539. DOI: 10.1021/acs.langmuir.5b02152.
(29) Marrink, S. J.; Risselada, H. J.; Yefimov, S.; Tieleman, D. P.; de Vries, A. H. The MARTINI Force Field: Coarse Grained Model for Biomolecular Simulations. *The Journal of Physical Chemistry B* **2007**, *111*, 7812-7824. Petersen, N.; Girard, M.; Riedinger, A.; Valsson, O. The Crucial Role of Solvation Forces in the Steric Stabilization of Nanoplatelets. *Nano Letters* **2022**, *22*, 9847–9853.
(30) Kumar, S.; Rosenberg, J. M.; Bouzida, D.; Swendsen, R. H.; Kollman, P. A. THE weighted histogram analysis method for free-energy calculations on biomolecules. I. The method. *Journal of Computational Chemistry* **1992**, *13* (8), 1011–1021. DOI: https://doi.org/10.1002/jcc.540130812.
